# Supplementary material for: Is echocardiography valid and reproducible in patients with atrial fibrillation? A systematic review
Source: Europace. 2017 Apr 6;19(9):1427–38. doi: 10.1093/europace/eux027 (PMC5834126; doi:10.1093/europace/eux027)
Supplement: Supplementary Table S1 [file supplementary_table_s1_eux027.pdf]

Europace

Is echocardiography valid and reproducible in patients with atrial fibrillation? A systematic review

--Manuscript Draft--

**Manuscript Number:**	EUPC-D-16-01168R1
**Full Title:**	Is echocardiography valid and reproducible in patients with atrial fibrillation? A systematic review
**Article Type:**	Clinical Research
**Keywords:**	atrial fibrillation; echocardiography; Reproducibility; Ejection fraction; heart failure; Diastolic.
**Corresponding Author:**	Dipak Kotecha, MBChB PhD MRCP FESC FHEA<br>University of Birmingham College of Medical and Dental Sciences<br>Birmingham, UNITED KINGDOM
**Corresponding Author Secondary Information:**	
**Corresponding Author's Institution:**	University of Birmingham College of Medical and Dental Sciences
**Corresponding Author's Secondary Institution:**	
**First Author:**	Dipak Kotecha, MBChB PhD MRCP FESC FHEA
**First Author Secondary Information:**	
**Order of Authors:**	Dipak Kotecha, MBChB PhD MRCP FESC FHEA
	Mohamed Mohamed
	Eduard Shantsila
	Bogdan A Popescu
	Richard P Steeds
**Order of Authors Secondary Information:**	
**Abstract:**	Aims: Echocardiography is vital in the routine assessment and management of atrial fibrillation (AF). We performed a systematic review of the validity and reproducibility of echocardiographic left ventricular systolic and diastolic function in AF, and optimal acquisition methods.<br>Methods: Online databases were searched for studies in patients with AF at the time of echocardiography (1960 to August 2015), prospectively registered with PROSPERO (CRD42015025297).<br>Results: The systematic review included 32 studies from 3,066 search results (1,968 patients with AF). Average age was 67 years, 33% were women, mean LVEF 53% (±10%) and average E/e' 11.7 (±2.7). Data on the validity and reproducibility of systolic indices were extremely limited. In contrast, diastolic parameters demonstrated correlation with invasive filling pressure and adequate reproducibility: E/e' (n=444) r= 0.47 to 0.79; IVRT (n=177) r= -0.70 to -0.95; E/Vp (n=55) r= 0.63 and 0.65; pulmonary vein diastolic flow (n=67) r= -0.80 and -0.91. Elevated E/e' (>15) was associated with functional capacity, quality of life and impaired prognosis. For optimal acquisition in AF patients, cardiac cycles with controlled heart rate (<100 beats/minute) and similar preceding and pre-preceding RR intervals are required. Cardiac cycle length and equivalence were more important that the number of beats averaged.<br>Conclusions: With careful selection of appropriate cardiac cycles, echocardiography is a valid tool to identify diastolic dysfunction in AF, and E/e' is an independent marker of clinical status and adverse prognosis. However, data on systolic function was extremely limited and requires further prospective study and assessment of variability in clinical practice.

Powered by Editorial Manager® and ProduXion Manager® from Aries Systems Corporation

Author Agreement Form (MUST be signed by all authors when paper is submitted)

Author Declaration Form

Title of Manuscript:

IS ECHOCARDIOGRAPHY VALID & REPRODUCIBLE IN PATIENTS WITH AF – A SYSTEMATIC REVIEW

This manuscript, or part of it, has neither been published nor is currently under consideration by any other Journal.

I declare that

1) **my co-authors listed below have read the manuscript and approved its submission to Europace. Each author has signed to that effect below, or**
2) **in the case of 5 or more authors, every author has read and approved the manuscript and has delegated me, as corresponding author, to sign this declaration on their behalf.**

Printed corresponding author name	Corresponding author signature
DIPAK KOTECHA	

Printed Co-author name	Co-author signature

Please wait...

If this message is not eventually replaced by the proper contents of the document, your PDF viewer may not be able to display this type of document.

You can upgrade to the latest version of Adobe Reader for Windows®, Mac, or Linux® by visiting http://www.adobe.com/go/reader_download.

For more assistance with Adobe Reader visit http://www.adobe.com/go/acrreader.

Windows is either a registered trademark or a trademark of Microsoft Corporation in the United States and/or other countries. Mac is a trademark of Apple Inc., registered in the United States and other countries. Linux is the registered trademark of Linus Torvalds in the U.S. and other countries.

Please wait...

If this message is not eventually replaced by the proper contents of the document, your PDF viewer may not be able to display this type of document.

You can upgrade to the latest version of Adobe Reader for Windows®, Mac, or Linux® by visiting http://www.adobe.com/go/reader_download.

For more assistance with Adobe Reader visit http://www.adobe.com/go/acrreader.

Windows is either a registered trademark or a trademark of Microsoft Corporation in the United States and/or other countries. Mac is a trademark of Apple Inc., registered in the United States and other countries. Linux is the registered trademark of Linus Torvalds in the U.S. and other countries.

Please wait...

If this message is not eventually replaced by the proper contents of the document, your PDF viewer may not be able to display this type of document.

You can upgrade to the latest version of Adobe Reader for Windows®, Mac, or Linux® by visiting http://www.adobe.com/go/reader_download.

For more assistance with Adobe Reader visit http://www.adobe.com/go/acrreader.

Windows is either a registered trademark or a trademark of Microsoft Corporation in the United States and/or other countries. Mac is a trademark of Apple Inc., registered in the United States and other countries. Linux is the registered trademark of Linus Torvalds in the U.S. and other countries.

Please wait...

If this message is not eventually replaced by the proper contents of the document, your PDF viewer may not be able to display this type of document.

You can upgrade to the latest version of Adobe Reader for Windows®, Mac, or Linux® by visiting http://www.adobe.com/go/reader_download.

For more assistance with Adobe Reader visit http://www.adobe.com/go/acrreader.

Windows is either a registered trademark or a trademark of Microsoft Corporation in the United States and/or other countries. Mac is a trademark of Apple Inc., registered in the United States and other countries. Linux is the registered trademark of Linus Torvalds in the U.S. and other countries.

Please wait...

If this message is not eventually replaced by the proper contents of the document, your PDF viewer may not be able to display this type of document.

You can upgrade to the latest version of Adobe Reader for Windows®, Mac, or Linux® by visiting http://www.adobe.com/go/reader_download.

For more assistance with Adobe Reader visit http://www.adobe.com/go/acrreader.

Windows is either a registered trademark or a trademark of Microsoft Corporation in the United States and/or other countries. Mac is a trademark of Apple Inc., registered in the United States and other countries. Linux is the registered trademark of Linus Torvalds in the U.S. and other countries.

Is echocardiography valid and reproducible in patients with atrial fibrillation? A systematic review (EUPC-D-16-01168)

Reviewer #1:

Authors did a great job with this systematic review. I have often discussions in daily clinical practice about the interpretation of diastolic function in the setting of AF.

Thank you for these comments. As you say, clinicians often have concerns about the interpretation of imaging tests in patients with AF, and yet the literature mostly relates to consensus opinion on the basis of small, selective studies. Our main aims were to clarify the current evidence-base, identify the key evidence gaps that limit clinical management of AF patients, and importantly, provide a platform for further research.

Thank you for your comments to improve our article. Please find a point-by-point reply below.

I have only minor comments:

1. Introduction: "The main objective was to assess the validity of echocardiographic measures against other modalities, and the reproducibility of parameters whilst in AF."

However, a lot of information is shared on other endpoints (wedge, exercise, BNP, etc). It is unclear whether the included articles are systematically checked for these secondary endpoints, including mortality, or that these information is shared because of the readability of the paper. In case of the former: do add the list of endpoints.

Thank you for this point. We systematically looked for any articles on both validity and reproducibility in patients with AF. The validity assessment included comparison with other modalities and the relationship with clinical events, which we report separately in the results section. The following amendments have been made to clarify this:

INTRODUCTION: We performed a systematic and focused review of published literature on the use of echocardiography for determination of systolic and diastolic LV function in patients with AF. Our main objectives ~~was~~ were to assess the validity of echocardiographic measures whilst in AF, both against other modalities and clinical outcomes, and the reproducibility of these parameters ~~whilst in AF~~. A further objective was to appraise the acquisition of images. This includes the optimal number of repeated measurements and cardiac cycle lengths that would reduce variability of systolic and diastolic evaluation and allow confidence in the ~~clinical~~ echocardiographic diagnosis of ~~heart failure~~systolic or diastolic dysfunction in AF.

METHODS: Data on validity (against other modalities and any relevant clinical associations) and reproducibility (both intra- and inter-observer variability) were extracted by three investigators independently (MM, ES and DK), and tabulated in a standardized data-extraction form.

2. In the line of the previous (p9 and further): "Another study by the same group identified no relationship between systolic parameters and a history of prior ischemic stroke in 330 patients with persistent AF and LVEF >40% 18, unlike the diastolic parameter E/e' (see below). These sentences are only relevant when these observations are part of the predefined study design. If not, one could ask whether it shouldn't be skipped...

Thank you – you are correct that this study was predominantly concerned with the association of diastolic parameters and prior stroke, so we have removed this sentence.

RESULTS: With respect to clinical outcomes, one study showed that LV systolic parameters were unrelated to exercise capacity in 73 stable AF patients [17]. ~~Another study by the same group identified no relationship between systolic parameters and a history of prior ischemic stroke in 330 patients with persistent AF and LVEF >40%, unlike the diastolic parameter E/e' (see below).~~ However, in a study of 196 patients with persistent AF, baseline GLS was independently associated with a composite of cardiovascular death…

3. invasive PCWP on right heart catheterization: is this an inclusion criterion for reviewing the article?

No – we were very broad in our search terms, using only "atrial fibrillation" and "echocardiography" and then manually searching for terms relating to any validity or reproducibility measures.

METHODS: Eligibility criteria & search strategy: All studies reporting validity or reproducibility data on LV systolic or diastolic function in AF patients were examined.

Reviewer #2:

The authors should be commended for this extensive review of the literature reporting on the validity and reproducibility of systolic and diastolic parameters of LV function in patients with AF.

Thank you for this comment, and those below to help us improve our article. Please find a point-by-point reply below.

As expected, the data are very limited, particularly for LV systolic measures, and the studies are retrospective (when it comes to prognosis analysis) which limit the validity of the meta-analysis. Basically, it is just a review of the literature. The authors also indicate the need for further studies evaluating the reproducibility of the LV systolic function measurements. The topic is timely. However, the scope of the study does not have many clinical implications: if we cannot control the rhythm and the heart rate, how can we assess LV systolic function? It is not really a limitation of the technique. It is more the implication of the clinical situation that makes difficult the reproducibility (and test-retest assessment).

The limited data on systolic function in AF is indeed an issue, particularly for clinical practice where echocardiography (and other imaging modalities) are frequently used and expected to be clinically valid in those with AF at the time of scanning. As demonstrated by a global AF registry, persistent and permanent AF make up over 70% of AF patients (Chiang et al; Circ Arrhythm EP 2012). Therefore, we believe that our systematic review does have important clinical implications:

1. **To highlight the lack of studies that compare echo to other modalities for estimation of systolic function whist in AF, and stimulate further research about how this can be achieved (see comment 1 below for further details).**
2. **To confirm that diastolic assessment is possible whilst in AF, and this has a moderate correlation with invasive wedge pressure (not dissimilar to sinus rhythm).**
3. **To optimize measurement by improving acquisition techniques that can lead to better validity and reproducibility of echo in AF. This includes not only a controlled heart rate (which is well known), but also the choice of cardiac cycles which could be easily implemented in routine clinical practice.**

Comments:

1.-What's new section: data on the validity and reproducibility of systolic indices in AF patients were extremely limited; this is a priority for future research. What do the authors expect from such research? I would be surprised if the results show that the measurements are more reproducible than in patients in sinus rhythm. Again, it is not a limitation of the technique. It is the clinical situation which limits the measurements.

Thank you for this important comment. Clinicians expect that the result of any cardiac imaging test is a good reflection of the current status of the patient, with which they can

base clinical decisions. Due to the variability in heart rate and stroke volume from beat-to-beat in patients with AF, it is likely that certain echocardiographic measures are more suited to AF, and that use of these will result in better correlation with clinical events and prognosis. Whilst we would not expect to reach similar reproducibility to sinus rhythm, choosing the correct method and optimizing acquisition are key priorities for future research. A number of centres in Europe are pursuing such research, including comparisons of LVEF assessment and speckle-tracking or strain in AF, and using automated acquisition to average hundreds of Doppler measurements sequentially. These new techniques could offer improvements in how we routinely apply echo in clinical practice. The following change has been made to the 'What's New' section:

'WHAT'S NEW': In this systematic review, data on the validity and reproducibility of systolic indices in AF patients were extremely limited; ~~this is a~~the best measure of systolic function and acquisition method in AF are prioritiesy for future research.

The following addition has been made to the discussion:

DISCUSSION: Whereas a properly acquired index-beat assessment approach, based on our data, should achieve good levels of validity and reproducibility for diastolic indices, the data on systolic parameters is clearly inadequate. It is unclear which measure of systolic function is best for patients who are scanned whilst in AF, and this should be a priority for future research. Although global strain at a low cut-off was associated with outcomes in one of the studies reviewed 32, more recent data suggests that the association of strain with mortality is attenuated in patients with AF and heart failure…

2.-" Measurement in AF is optimised when the two preceding cardiac cycles have similar RRintervals and the heart rate is controlled (<100 beats/minute)." Measurement of what? Can the authors finalize the sentence?

Thanks for noting this omission. The sentence has been amended:

'WHAT'S NEW': Measurement of systolic and diastolic function in AF is optimised when the two preceding cardiac cycles have similar RR-intervals and the heart rate is controlled (<100 beats/minute).

3.-" All of these important clinical decisions are based on the assumption that echocardiographic measures are as valid and reproducible in AF as they are in sinus rhythm". This is an act of faith. Who believes this? This statement is not correct. And again it is not the limitation of the technique.

Very true! The sentence has been amended:

INTRODUCTION: All of these important clinical decisions ~~are based on the assumption that~~ require echocardiographic measures that are ~~as~~ valid and reproducible, regardless of cardiac rhythm ~~in AF as they are in sinus rhythm~~.

4.-An important limitation of the meta-analysis is the observational design of the included studies. That means that it has 0 implications in clinical practice and future recommendations. The authors should avoid the term meta-analysis and just say review article.

Thank you – we have been very clear (as you rightly say) that this is a systematic review and NOT a meta-analysis:

TITLE: Is echocardiography valid and reproducible in patients with atrial fibrillation? A systematic review (as per PRISMA guidelines, 'meta-analysis' has been omitted)

METHODS: Meta-analysis of comparative data between AF and sinus rhythm was not possible…

LIMITATIONS: We were unable to perform meta-analysis, not only because of the lack of published standard deviations for validation and reproducibility measures, but also...

5.-There is only 1 study evaluating LV global longitudinal strain. If the authors want to keep the word meta-analysis, I would remove this study. The same concerns for myocardial performance index (actually this parameter is similar to the Tei index and it is well known that in patients with AF is not valid).

Thank you – please note response to comment 4 that our study is not a meta-analysis. In a systematic review, all references that fit the inclusion and exclusion criteria must be commented on. To remove any studies based on preconception of lack of validity in AF would not be appropriate and contrary to our prospectively-registered protocol

(see http://www.crd.york.ac.uk/PROSPERO/display_record.asp?ID=CRD42015025297).

6.-what are the clinical implications of the present analysis and how do the authors think that further studies will change the management of AF patients? Because I do not see it. This is an important group of patients that will increase the pool pf patients with HFPEF.

Please see the three points made on page 3 as to the clinical importance of our study. Currently, very few echocardiographers or clinical reviewers are performing echocardiography in AF with attention to cardiac cycle length. Many centres assume

systolic parameters are valid, whilst discounting E/e' measurement in AF patients and relying on parameters we have shown to be less reproducible or less valid in respect to other modalities or clinical outcomes. Routine application of the results of our findings has the potential to improve the diagnostic value of echocardiography in AF patients.

The following change has been made to the beginning of the discussion:

---

*DISCUSSION: The main findings of this systematic review were that diastolic indices, in particular E/e', were valid and reproducible in patients with AF, whereas data for systolic parameters were extremely limited. We also identified consensus amongst numerous studies that the optimal acquisition of echocardiography in AF patients occurred when preceding and pre-preceding cycle lengths are equivalent, rather than according to the number of repeated measurements taken. These findings have important clinical impact, dispelling preconceptions about the utility of diastolic variables ~~in patients with AF~~, ~~and~~ highlighting key areas in need of further prospective study, and improving the diagnostic value of echocardiography in patients with AF (**Figure 3**).*

We have also added the word 'selective' to the conclusion to make the point clear that these studies have selection biases.

CONCLUSION: In selected patients with atrial fibrillation, diastolic echocardiographic parameters have been validated against invasive filling pressure, and E/e' is an independent marker of functional impairment and adverse prognosis.

Is echocardiography valid and reproducible in patients with atrial fibrillation? A systematic review

What's New? (113 words)

1. The new 2016 ESC Guidelines on AF recommend echocardiography in all AF patients to guide management (I C).
2. In this systematic review, data on the validity and reproducibility of systolic indices in AF patients were extremely limited; the best measure of systolic function and acquisition method in AF are priorities for future research.
3. Diastolic parameters in AF have been validated against invasive filling pressure with adequate reproducibility. Elevated E/e' (>15) is also associated with functional capacity, quality of life and impaired prognosis.
4. Measurement of systolic and diastolic function in AF is optimised when the two preceding cardiac cycles have similar RR-intervals and the heart rate is controlled (<100 beats/minute).

Is echocardiography valid and reproducible in patients with atrial fibrillation? A systematic review

Abstract

Aims: Echocardiography is vital in the routine assessment and management of atrial fibrillation (AF). We performed a systematic review of the validity and reproducibility of echocardiographic left ventricular systolic and diastolic function in AF, and optimal acquisition methods.

Methods: Online databases were searched for studies in patients with AF at the time of echocardiography (1960 to August 2015), prospectively registered with PROSPERO (CRD42015025297).

Results: The systematic review included 32 studies from 3,066 search results (1,968 patients with AF). Average age was 67 years, 33% were women, mean LVEF 53% (±10%) and average E/e’ 11.7 (±2.7). Data on the validity and reproducibility of systolic indices were extremely limited. In contrast, diastolic parameters demonstrated correlation with invasive filling pressure and adequate reproducibility: E/e’ (n=444) r= 0.47 to 0.79; IVRT (n=177) r= -0.70 to -0.95; E/Vp (n=55) r= 0.63 and 0.65; pulmonary vein diastolic flow (n=67) r= -0.80 and -0.91. Elevated E/e’ (>15) was associated with functional capacity, quality of life and impaired prognosis. For optimal acquisition in AF patients, cardiac cycles with controlled heart rate (<100 beats/minute) and similar preceding and pre-preceding RR intervals are required. Cardiac cycle length and equivalence were more important that the number of beats averaged.

Conclusions: With careful selection of appropriate cardiac cycles, echocardiography is a valid tool to identify diastolic dysfunction in AF, and E/e' is an independent marker of clinical status and adverse prognosis. However, data on systolic function was extremely limited and requires further prospective study and assessment of variability in clinical practice.

Key Words: Atrial fibrillation; Echocardiography; Reproducibility; Ejection fraction; Heart failure; Diastolic.

Is echocardiography valid and reproducible in patients with atrial fibrillation? A systematic review

Condensed abstract (50 words)

Echocardiography is recommended in all AF patients, but validity and reproducibility is unclear. In this systematic review, we identified a lack of data on systolic function, whereas diastolic parameters have been validated against invasive pressure with adequate reproducibility. Optimal acquisition requires the two preceding cardiac cycles to have similar RR-intervals.

UNIVERSITY OF BIRMINGHAM

Institute of Cardiovascular Sciences
University of Birmingham
The Medical School, Vincent Drive, Edgbaston, B15 2TT

27th December 2016

Dear Prof. Camm & Editorial Team

Europace

Thank you for considering our article, titled “Is echocardiography valid and reproducible in patients with atrial fibrillation? A systematic review”.

Please find enclosed a detailed point-by-point response to the reviewer comments, a manuscript file with tracked changes and a clean manuscript copy.

I confirm that references are in Europace format and that the Europace guidelines version is used. Figures are supplied in eps format (Illustrator originals and CMYK tiff images are also available if required).

Thank you for the opportunity to improve our manuscript.

Best wishes for 2017. Yours sincerely,

Dipak Kotecha, MBChB, PhD, PCAP, MRCP, FESC, FHEA
National Institute for Health Research (NIHR) Career Development Fellow
Clinician Scientist in Cardiovascular Medicine, University of Birmingham, UK
Honorary Consultant Cardiologist, University Hospitals Birmingham NHS Trust, UK
Adjunct Research Fellow, Monash University, Melbourne, Australia
European Society of Cardiology (ESC) Guideline Taskforce for Atrial Fibrillation & ESC Education Committee

Manuscript TRACKED

Click here to download Manuscript (incl.refs, Acknowledgments, Funding, Ethics, etc follow guidelines)

Title: Is echocardiography valid and reproducible in patients with atrial fibrillation? A systematic review

Brief Title: Echocardiography in atrial fibrillation

Authors: Dipak Kotecha[1,2,3*], Mohamed Mohamed[4], Eduard Shantsila[1], Bogdan A Popescu[5], Richard P Steeds[1,3]

From the (1) University of Birmingham Institute of Cardiovascular Sciences, Birmingham, UK; (2) Centre of Cardiovascular Research and Education in Therapeutics, Monash University, Melbourne, Australia; (3) University Hospitals Birmingham NHS Trust, Birmingham, UK; (4) East Kent Hospitals University NHS Trust, Ashford, UK; and (5) University of Medicine and Pharmacy 'Carol Davila' - Euroecolab, and Institute of Cardiovascular Diseases 'Prof. C. C. Iliescu' Bucharest, Romania.

*Address for correspondence:
Dr Dipak Kotecha MBChB PhD MRCP FESC FHEA
University of Birmingham Institute of Cardiovascular Sciences,
The Medical School, Vincent Drive, Edgbaston, Birmingham, B15 2TT, United Kingdom.
Email: d.kotecha@bham.ac.uk
Tel: +44 121 507 5080 Fax: +44 121 554 4083

Word count (abstract): 249
Word count (text, excl. references): ~~3372~~3387

Key Words: Atrial fibrillation; Echocardiography; Reproducibility; Ejection fraction; Heart failure; Diastolic.

Abstract

Aims: Echocardiography is vital in the routine assessment and management of atrial fibrillation (AF). We performed a systematic review of the validity and reproducibility of echocardiographic left ventricular systolic and diastolic function in AF, and optimal acquisition methods.

Methods: Online databases were searched for studies in patients with AF at the time of echocardiography (1960 to August 2015), prospectively registered with PROSPERO (CRD42015025297).

Results: The systematic review included 32 studies from 3,066 search results (1,968 patients with AF). Average age was 67 years, 33% were women, mean LVEF 53% (±10%) and average E/e' 11.7 (±2.7). Data on the validity and reproducibility of systolic indices were extremely limited. In contrast, diastolic parameters demonstrated correlation with invasive filling pressure and adequate reproducibility: E/e' (n=444) r= 0.47 to 0.79; IVRT (n=177) r= -0.70 to -0.95; E/Vp (n=55) r= 0.63 and 0.65; pulmonary vein diastolic flow (n=67) r= -0.80 and -0.91. Elevated E/e' (>15) was associated with functional capacity, quality of life and impaired prognosis. For optimal acquisition in AF patients, cardiac cycles with controlled heart rate (<100 beats/minute) and similar preceding and pre-preceding RR intervals are required. Cardiac cycle length and equivalence were more important that the number of beats averaged.

Conclusions: With careful selection of appropriate cardiac cycles, echocardiography is a valid tool to identify diastolic dysfunction in AF, and E/e' is an independent marker of clinical status and adverse prognosis. However, data on systolic function was extremely limited and requires further prospective study and assessment of variability in clinical practice.

Condensed abstract (50 words)

Echocardiography is recommended in all AF patients, but validity and reproducibility is unclear. In this systematic review, we identified a lack of data on systolic function, whereas diastolic parameters have been validated against invasive pressure with adequate reproducibility. Optimal acquisition requires the two preceding cardiac cycles to have similar RR-intervals.

What's New? (~~99~~ 113 words)

1. The new 2016 ESC Guidelines on AF recommend echocardiography in all AF patients to guide management (I C).
2. In this systematic review, data on the validity and reproducibility of systolic indices in AF patients were extremely limited; ~~this is a~~the best measure of systolic function and acquisition method in AF are prioritiesy for future research.
3. Diastolic parameters in AF have been validated against invasive filling pressure with adequate reproducibility. Elevated E/e' (>15) is also associated with functional capacity, quality of life and impaired prognosis.
4. Measurement of systolic and diastolic function in AF is optimised when the two preceding cardiac cycles have similar RR-intervals and the heart rate is controlled (<100 beats/minute).

Introduction

Atrial fibrillation (AF) is an increasingly common heart rhythm disturbance that leads to frequent hospital admissions, heart failure, stroke, and higher mortality.[1] There is a close relationship between AF and heart failure, with numerous risk factors common to both conditions and shared pathophysiology in patients with both reduced [2] and preserved [3] left ventricular ejection fraction (LVEF). Depending on the type of AF, the rate of prevalent heart failure is between 33% and 56% [4]; hence clinicians treating patients with AF need reliable information on both systolic and diastolic left ventricular (LV) function. Echocardiography is the primary tool used in clinical practice and provides vital guidance to determine appropriate use of anticoagulation, rate-control therapy and rhythm-control strategies, as well as important information on co-existing or precipitating pathology and prognostic data [5]. All of these important clinical decisions ~~are based on the assumption that~~require echocardiographic measures that are ~~as~~ valid and reproducible, regardless of cardiac rhythm ~~in AF as they are in sinus rhythm~~.

The loss of synchronized atrial contraction and altered left atrial pressure is likely to affect the reproducibility of echocardiographic measurements in AF. Factors that have been implicated include the ratio of preceding to pre-preceding cycle length and heart rate during image acquisition. Both of these influence the volume of ejection and consequently the results of the most commonly-used measurements of LV function, particularly where these are taken over a number of cardiac cycles. Joint guidelines published by the American Society of Echocardiography and the European Association of Cardiovascular Imaging suggest a minimum of five beats in AF patients, although this is based on consensus opinion [6]. For diastolic function, the British Society of Echocardiography recommends averaging over 5-10 beats during cycle lengths equivalent to a heart rate between 60-80 beats/minute.

We performed a systematic and focused review of published literature on the use of echocardiography for determination of systolic and diastolic LV function in patients with AF. Our main objectives was were to assess the validity of echocardiographic measures whilst in AF, both against other modalities and clinical outcomes, and the reproducibility of these parameters whilst in AF. A further objective was to appraise the acquisition of images. This includes the optimal number of repeated measurements and cardiac cycle lengths that would reduce variability of systolic and diastolic evaluation and allow confidence in the clinical echocardiographic diagnosis of heart failuresystolic or diastolic dysfunction in AF.

Methods

Eligibility criteria & search strategy

All studies reporting validity or reproducibility data on LV systolic or diastolic function in AF patients were examined. There was no restriction on study design, however only adult populations with AF at the time of echocardiography were considered. Exclusion criteria included case reports, animal studies and studies that were only published in abstract form or in a language other than English. All editorials, commentaries and informal reviews of other literature were also excluded, as were studies only assessing left atrial size or function. An online search was performed of PubMed and the Cochrane library (inception to December 2014, and then extended to August 2015), including the broad terms "atrial fibrillation" and "echocardiography" using MESH headings and title/abstract searches, including syntax variations. We also conducted manual screening of relevant reviews and reference lists. The systematic review was reported according to the Preferred Reporting Items for Systematic reviews and Meta-Analyses (PRISMA) guidelines and prospectively registered with the PROSPERO database of systematic reviews (http://www.crd.york.ac.uk/PROSPERO/display_record.asp?ID=CRD42015025297).

Outcomes

The primary outcomes of interest were echocardiographic measures of LV systolic and diastolic function. For systolic function, these included LVEF using biplane Simpson's method or 3D volume assessment and measurement of strain (peak longitudinal systolic strain [PLSS] and global longitudinal strain [GLS]). For diastolic function, we included assessment of isovolumic relaxation time (IVRT), mitral E-wave deceleration time, the ratio of mitral peak E velocity to tissue Doppler early diastolic velocity e' (E/e'), pulmonary venous (PV) flow diastolic

deceleration time (PVd-DT), and the ratio of mitral peak E velocity to the velocity of diastolic flow propagation measured with colour Doppler M-mode (E/Vp). For all parameters, we extracted data on validity against other modalities (for example, pulmonary capillary wedge pressure [PCWP] on right heart catheterization) and estimates of intra and inter-operator reproducibility. We also noted the method by which studies collected data, including the number of repeated measures and cardiac cycle lengths. A secondary outcome was to record average values of echocardiographic measures in AF, for comparison with published norms in patients with sinus rhythm.

Data collection and quality assessment

Data on validity (against other modalities and any relevant clinical associations) and reproducibility (both intra- and inter-observer variability) were extracted by three investigators independently (MM, ES and DK), and tabulated in a standardized data-extraction form. Study quality was assessed using the Risk of Bias Assessment Tool for Non-randomized Studies (RoBANS), which addresses selection bias, exposure measurement, blinding, the completeness of outcome data and selectivity of reporting [7]. Risk of bias was assessed by two investigators independently (MM and ES) and discrepancies resolved by group discussion and additional adjudication (DK).

Data synthesis and statistical analysis

Baseline demographics were pooled from all studies providing suitable data (including variance where applicable), and are summarized as a weighted mean according to sample size. Outcomes were synthesized qualitatively. Meta-analysis of comparative data between AF and sinus rhythm was not possible due to the limited studies available and a lack of published data on the variance of outcome measures. Analyses were performed on Stata Version 14.1 (StataCorp LP,

Texas).

Results

The search strategy identified a total of 3,066 records of which 2,945 were excluded, primarily due to lack of relevance to echocardiography in AF, and a further 89 excluded after full text review (**Figure 1**). Thirty-two observational studies were included in the final review [8-39], the majority of which were single-centre studies. **Table 1** highlights the populations examined and the key findings relating to patients with AF. There was marked heterogeneity in the type of AF (paroxysmal, persistent or permanent), heart failure status, LVEF and clinical demographics. The weighted-average age was 66.9 years and a third were women (**Table 2**). Overall, studies recorded a mean LVEF of 52.5% and average E/e' of 11.7 in AF. Heart rate was usually below 80 beats/minute, with a minority of studies excluding patients above a specific heart rate target (typically >100 beats/minute). Many studies excluded patients with AF due to valvular heart disease. Only four studies enrolled 100 or more patients, and there were frequent references to selecting participants with adequate quality echocardiographic images. As a result, the risk of bias for selection and blinding were universally high, although in other domains, the risk of bias was more variable (**Supplementary Table A**).

Systolic function: Validity and reproducibility

Data for the validity of systolic function indices in patients with AF were extremely limited. We found no external validation studies (for example, comparing results with other modalities such as cardiac magnetic resonance or nuclear imaging). There were however examples of within-study or internal validation (such as correlation of new 3D techniques with conventional biplane Simpsons, or strain with LVEF) [9, 33]. With respect to clinical outcomes, one study showed that

LV systolic parameters were unrelated to exercise capacity in 73 stable AF patients [17]. ~~Another study by the same group identified no relationship between systolic parameters and a history of prior ischemic stroke in 330 patients with persistent AF and LVEF >40%, unlike the diastolic parameter E/e' (see below).~~ However, in a study of 196 patients with persistent AF, baseline GLS was independently associated with a composite of cardiovascular death, non-fatal stroke and heart failure hospitalization after 21 (±10) months follow-up [32]. This relationship persisted in multivariate analysis (hazard ratio 1.12, 95% CI 1.02-1.23, p=0.014), whereas LVEF and other measures of systolic function were not independently significant. The optimal, post-hoc defined GLS cut-off for predicting event-free survival was -12.5%, and this incrementally added to clinical predictors of adverse outcome [32].

Reproducibility of systolic function indices are summarized in **Table 3**. A wide array of study and acquisition methods made data synthesis unfeasible, however reproducibility was reasonable in AF patients using single-beat methods [19, 29, 31, 32, 39]. One study examining AF patients with irregularity on their electrocardiogram found that to achieve similar variability for cardiac output in AF as with sinus rhythm, three times the number of beats were required (13 versus 4 beats, respectively) [12]. In contrast, although there was higher inter-observer variability for 3D-LVEF using conventional 4-beat acquisition in AF compared to sinus rhythm (17.9% versus 3.9%, respectively), when using single-beat acquisition, reproducibility was similar regardless of heart rhythm (5.6% in AF, versus 4.5% in sinus rhythm) [29].

Diastolic function: Validity and reproducibility

Considerably more data were available for the use of diastolic parameters in AF (**Table 4**). Twenty studies provided correlations with invasive PCWP on right heart catheterization for a range of diastolic indices. IVRT was assessed in 4 studies (n=177) and inverse correlations with

PCWP were all highly statistically significant, ranging from -0.70 to -0.95 [11, 22, 34, 35]. Seven studies examined mitral deceleration time (n=324), of which 2 found no correlation with PCWP [27, 30] and 5 identified moderate inverse correlation [10, 21, 22, 34, 35]. All 5 studies of E/e' (n=444) showed significant association with PCWP, ranging from 0.47 to 0.79, and including e' derived from both septal and lateral positions [15, 20, 27, 30, 36]. Using a dual Doppler method, the combination of E/e' and the time between E and e' (cut-points at >14.6 and >34ms respectively), improved the sensitivity and specificity for predicting elevated PCWP versus either alone [36]. Compared to those in sinus rhythm, AF patients demonstrated a similar correlation with PCWP for the ratio of IVRT to time between E and e' in patients with mitral valve disease [11]. E/Vp and the deceleration time of PV diastolic flow were each assessed in 2 studies (n=55 and n=67, respectively) and both parameters showed a high degree of correlation with PCWP [10, 21, 22, 24]. Diastolic PV flow was better than mitral indices for estimating PCWP in one study of 35 AF patients [10].

In regard to clinical outcomes, a retrospective analysis of 230 AF patients identified that septal E/e' >15 was independently associated with mortality during follow-up of 245 (± 200) days, both in patients with impaired and preserved LVEF [23]. Deceleration time <150 ms was associated with mortality during follow-up of 25 (± 11) months in AF patients with LVEF <40% who had been hospitalized for heart failure, with a similar impact in AF patients (n=40) as those with sinus rhythm (n=100) [25]. Diastolic indices, including E/e' and E/Vp, have also been shown to correlate with B-type natriuretic peptide (BNP), a biomarker strongly associated with adverse prognosis [15, 24]. E/e' was the only echocardiographic variable of LV function related to exercise capacity in 73 patients with AF (multivariate adjusted coefficient β= -0.12; p= 0.032) [17]. The same group also showed in one of the only multicentre studies that septal E/e' was associated with prior ischaemic stroke in 330 AF patients with LVEF >40% (adjusted odds ratio 1.21, 95%

CI 1.08-1.37; p=0.002), unlike clinical and echocardiographic parameters such and age, BNP, or LVEF [18]. E/e' also correlates with 6-minute walk distance and quality of life, as seen in a retrospective study of 48 patients with AF and preserved LVEF [26].

Reproducibly of diastolic indices is summarized in **Table 4**, with intra- and inter-observer mean differences, coefficients of variation and test-retest variability reasonable in the majority of the 23 studies (n=997) [10, 13, 15, 20-27, 34-36]. Of note, E/e' was shown to be reliable when measured one week apart (correlation coefficient 0.87, p<0.05) [26], and the variability of diastolic indices was similar in AF and sinus rhythm in a small cohort of patients from the Framingham study [13].

Acquisition: Cycle length and cycle repeats

The irregular RR interval in AF has led to concern about the reliability of both systolic and diastolic measures, and there is clinical uncertainty about the number of repeated measures required and optimal cycle length. Historical data have shown that the RR interval affects LVEF in AF patients, more so than in sinus rhythm [8]. More recent studies have confirmed that the cycle length of preceding RR intervals in AF is strongly related to stroke volume [37]. LV ejection velocity is lower when pre-preceding RR intervals are longer, and differences in systolic performance are minimized when the preceding and pre-preceding RR interval lengths are similar [28]. Beat-to-beat variability in stroke volume increases as heart rate increases in AF patients [40], and the effect of preceding and pre-preceding RR intervals on stroke volume is most pronounced at higher heart rates [14].

With regard to the number of repeated measurements required, when preceding and pre-preceding RR interval lengths are equivalent (<60ms difference), measurement of PLSS in patients with persistent or permanent AF was similar using a single index-beat, as compared to averaging 15 cardiac cycles (r=0.97, p<0.001) [19]. In another study, index-beat assessment gave

similar values to 10-second averages for myocardial strain and strain rate (r=0.94, p<0.001) [16]. The benefit of averaging a number of beats with similar preceding and pre-preceding RR intervals and with cycle lengths of 500ms or greater was confirmed in two further studies [38, 39]. Using 3D volume datasets, a single-beat measurement in AF had lower variability than conventional 4-beat acquisition [29], although whether a single-beat analysis has the same association with clinical outcomes is currently unknown. For diastolic function, retest variability of E/e' was similar over 10 or 50 cardiac cycles in AF patients with preserved LVEF [20]. In another study of post-operative AF patients, the correlation of E/e' to PCWP was no different when sampling over 10 beats or in a single cycle with the longest RR interval (r= 0.47 and 0.44, respectively) [27].

These results suggest that choosing appropriate cardiac cycles with similar RR interval is more important than the absolute number of cycles measured (**Figure 2**). Of clinical importance, Nagueh *et al.* found less Doppler variability in patients at higher LV filling pressure [22], suggesting that measurement error might actually be reduced in those patients at the highest risk of adverse events.

Discussion

The main findings of this systematic review were that diastolic indices, in particular E/e’, were valid and reproducible in patients with AF, whereas data for systolic parameters were extremely limited. We also identified consensus amongst numerous studies that the optimal acquisition of echocardiography in AF patients occurred when preceding and pre-preceding cycle lengths are equivalent, rather than according to the number of repeated measurements taken. These findings have important clinical impact, dispelling preconceptions about the utility of diastolic variables ~~in patients with AF~~, ~~and~~ highlighting key areas in need of further prospective study, and improving the diagnostic value of echocardiography in patients with AF (**Figure 3**).

Echocardiography is a vital part of the assessment of AF patients, and is now recommend in all AF patients to guide management (class I, level of evidence C).[1] Numerous narrative reviews have been published concerning both systolic and diastolic function, however, this is the first systematic assessment of the validity and reproducibility of measurements. Echocardiography is an important component of initial management and is cost-effective for newly diagnosed patients with AF [41]. Knowledge about the type of heart failure in AF (preserved or reduced ejection fraction), has an important bearing on prognosis [42]. Identifying reduced LVEF also has consequences for the choice of rate- and rhythm-control therapy, for example the choice of beta-blockers or digoxin [43, 44], and the avoidance of non-dihydropyridine calcium channel blockers and class I anti-arrhythmic drugs. Echocardiography is also vital for the planning and follow-up of patients undergoing catheter, surgical and hybrid ablation for AF, as well as left atrial appendage closure.

As all of the studies were undertaken on patients in AF, the pooled data gives clinical guidance as to expected average values. The weighted-mean LVEF was 52.5%, and although a number of

studies either excluded or only enrolled those with heart failure, this was similar to the RealiseAF Global Registry (LVEF 54.3% in persistent and 53.3% in permanent AF) [4]. E/e' values were consistently higher than seen in 103 healthy volunteers (lateral E/e' 6.2±1.8 in age-range 60-69 years) [45] but similar to 100 sinus rhythm patients undergoing coronary angiography [46] and 951 sinus rhythm patients with isolated diastolic dysfunction and e'/a' <1. [47] However, even though average estimates are likely to be higher in AF patients (with associated comorbidities) than sinus rhythm, the cut-off value of E/e' >15 was still a good marker of adverse events and functional capacity in AF. Validation of E/e' against invasive filling pressure was reasonable in AF, and similar to correlation values published in sinus rhythm. For sinus rhythm, this includes lateral E/e′ r= 0.51 in 100 patients, lateral E/e' r= 0.86 in 100 patients, and septal E/e' r= 0.46 in 60 echocardiogram studies in 15 patients. [46, 48, 49] However, a recent systematic review of E/e' in sinus rhythm identified concerns over reliability of this parameter to estimate LV filling pressure.[50]

In all cases, there is the assumption that echocardiographic parameters are reliable in AF, despite the irregular ejection and rate. We have shown that stroke volume and LVEF do vary according to cycle length, particularly in respect to the RR intervals preceding measurement. In contrast to sinus rhythm, echocardiographers need to carefully appraise how and when to acquire measurements in order to accurately identify LV dysfunction in AF patients. Simultaneous assessment of both E and e' are now available in order to provide a single-beat analysis of E/e' (dual Doppler method). There are theoretical advantages to this process in reducing error, particularly in AF where successive beats are likely to vary. The dual Doppler method appears to offer better validation versus invasive PCWP (see **Table 4**), and in one study conferred a smaller amount of variability in E/e' between operators (7.1% versus 13.4% using conventional analysis over 10 cycles) [20]. However, it is unclear if this has any advantage over

properly acquired index-beat assessment, and availability in clinical practice is currently limited. Whereas a properly acquired index-beat assessment approach, based on our data, should achieve good levels of validity and reproducibility for diastolic indices, the data on systolic parameters is clearly inadequate. It is unclear which measure of systolic function is best for patients who are scanned whilst in AF, and this should be a priority for future research. Although global strain at a low cut-off was associated with outcomes in one of the studies reviewed [32], more recent data suggests that the association of strain with mortality is attenuated in patients with AF and heart failure with reduced LVEF (p value for interaction = 0.036) [51]. Further prospective studies, either in the context of controlled trials [52] or in routine clinical practice, are urgently needed to support the large volume of echocardiograms performed in patients with AF. As clinicians, we also need to know the minimum number of index beats required to maintain equivalence but reduce the time required for scanning, and for confirmation of reproducibility at different heart rates and grades of systolic and diastolic LV dysfunction.

Study limitations

There are numerous limitations to our review, most notably the risk of bias, particularly selection and blinding bias, as patients were often selected on the basis of echocardiogram quality. However, this is no different to studies in sinus rhythm. There are likely to be other studies assessing the reproducibility of echo parameters in AF, missed by our systematic search if reproducibility was not listed as a major outcome. We were unable to perform meta-analysis, not only because of the lack of published standard deviations for validation and reproducibility measures, but also the heterogeneity of populations assessed. Although most studies made reference to 'chronic AF', the duration and type of AF was often not disclosed. Most of the studies excluded valve disease (with differing definitions) and there was limited data above a heart rate of 100 beats/min. Finally, considering the importance of diagnosing heart failure in

patients with AF, and how common these conditions are in clinical practice, the relatively small number of studies identified in this systematic review is a surprising limitation, and one that requires further attention.

Conclusions

In selected patients with atrial fibrillation, diastolic echocardiographic parameters have been validated against invasive filling pressure, and E/e’ is an independent marker of functional impairment and adverse prognosis. Averaging single-beat assessments are reproducible and should be acquired in cycles with similar preceding length and controlled heart rate. However, data on the validity and reproducibility of systolic indices are extremely limited. Considering the importance of heart failure and assessment of systolic function in AF, further assessment of variability in routine clinical practice is urgently needed.

References

1. Kirchhof P, Benussi S, Kotecha D, Ahlsson A, Atar D, Casadei B, *et al.* 2016 ESC Guidelines for the management of atrial fibrillation developed in collaboration with EACTS. *Europace*. 2016;**18**:1609-1678

2. Kotecha D, Piccini JP. Atrial fibrillation in heart failure: what should we do? *Eur Heart J*. 2015;**36**:3250-3257

3. Kotecha D, Lam CS, Van Veldhuisen DJ, Van Gelder IC, Voors AA, Rienstra M. Heart Failure With Preserved Ejection Fraction and Atrial Fibrillation: Vicious Twins. *J Am Coll Cardiol*. 2016;**68**:2217-2228

4. Chiang CE, Naditch-Brule L, Murin J, Goethals M, Inoue H, O'Neill J, *et al.* Distribution and risk profile of paroxysmal, persistent, and permanent atrial fibrillation in routine clinical practice: insight from the real-life global survey evaluating patients with atrial fibrillation international registry. *Circ Arrhythm Electrophysiol*. 2012;**5**:632-639

5. Donal E, Lip GY, Galderisi M, Goette A, Shah D, Marwan M, *et al.* EACVI/EHRA Expert Consensus Document on the role of multi-modality imaging for the evaluation of patients with atrial fibrillation. *Eur Heart J Cardiovasc Imaging*. 2016;**17**:355-383

6. Lang RM, Badano LP, Mor-Avi V, Afilalo J, Armstrong A, Ernande L, *et al.* Recommendations for cardiac chamber quantification by echocardiography in adults: an update from the American Society of Echocardiography and the European Association of Cardiovascular Imaging. *Eur Heart J Cardiovasc Imaging*. 2015;**16**:233-270

7. Kim SY, Park JE, Lee YJ, Seo HJ, Sheen SS, Hahn S, *et al.* Testing a tool for assessing the risk of bias for nonrandomized studies showed moderate reliability and promising validity. *J Clin Epidemiol*. 2013;**66**:408-414

8. Belenkie I. Beat-to-beat variability of echocardiographic measurements of left ventricular end diastolic diameter and performance. *J Clin Ultrasound*. 1979;**7**:263-268

9. Benyounes N, Lang S, Soulat-Dufour L, Obadia M, Gout O, Chevalier G, *et al.* Can global longitudinal strain predict reduced left ventricular ejection fraction in daily echocardiographic practice? *Arch Cardiovasc Dis*. 2015;**108**:50-56

10. Chirillo F, Brunazzi MC, Barbiero M, Giavarina D, Pasqualini M, Franceschini-Grisolia E, *et al.* Estimating mean pulmonary wedge pressure in patients with chronic atrial fibrillation from transthoracic Doppler indexes of mitral and pulmonary venous flow velocity. *J Am Coll Cardiol*. 1997;**30**:19-26

11. Diwan A, McCulloch M, Lawrie GM, Reardon MJ, Nagueh SF. Doppler Estimation of Left Ventricular Filling Pressures in Patients With Mitral Valve Disease. *Circulation*. 2005;**111**:3281-3289

12. Dubrey SW, Falk RH. Optimal number of beats for the Doppler measurement of cardiac output in atrial fibrillation. *J Am Soc Echocardiogr*. 1997;**10**:67-71

13. Galderisi M, Benjamin EJ, Evans JC, D'Agostino RB, Fuller DL, Lehman B, *et al.* Intra- and interobserver reproducibility of Doppler-assessed indexes of left ventricular diastolic function in a population-based study (the Framingham Heart Study). *Am J Cardiol*. 1992;**70**:1341-1346

14. Kerr AJ, Simmonds MB, Stewart RA. Influence of heart rate on stroke volume variability in atrial fibrillation in patients with normal and impaired left ventricular function. *Am J Cardiol*. 1998;**82**:1496-1500

15. Kusunose K, Yamada H, Nishio S, Tomita N, Niki T, Yamaguchi K, *et al.* Clinical utility of single-beat E/e' obtained by simultaneous recording of flow and tissue Doppler velocities in atrial fibrillation with preserved systolic function. *JACC Cardiovasc Imaging*. 2009;**2**:1147-1156

16. Kusunose K, Yamada H, Nishio S, Tomita N, Hotchi J, Bando M, *et al.* Index-beat assessment of left ventricular systolic and diastolic function during atrial fibrillation using myocardial strain and strain rate. *J Am Soc Echocardiogr*. 2012;**25**:953-959

17. Lee SH, Jung JH, Choi SH, Lee N, Oh DJ, Ryu KH, *et al.* Exercise intolerance in patients with atrial fibrillation: clinical and echocardiographic determinants of exercise capacity. *J Am Soc Echocardiogr*. 2005;**18**:1349-1354

18. Lee SH, Choi S, Chung WJ, Byun YS, Ryu SK, Pyun WB, *et al.* Tissue Doppler index, E/E', and ischemic stroke in patients with atrial fibrillation and preserved left ventricular ejection fraction. *J Neurol Sci*. 2008;**271**:148-152

19. Lee CS, Lin TH, Hsu PC, Chu CY, Lee WH, Su HM, *et al.* Measuring left ventricular peak longitudinal systolic strain from a single beat in atrial fibrillation: validation of the index beat method. *J Am Soc Echocardiogr*. 2012;**25**:945-952

20. Li C, Zhang J, Zhou C, Huang L, Tang H, Rao L. Will simultaneous measurement of E/e' index facilitate the non-invasive assessment of left ventricular filling pressure in patients with non-valvular atrial fibrillation? *Eur J Echocardiogr*. 2010;**11**:296-301

21. Matsukida K, Kisanuki A, Toyonaga K, Murayama T, Nakashima H, Kumanohoso T, *et al.* Comparison of transthoracic Doppler echocardiography and natriuretic peptides in predicting mean pulmonary capillary wedge pressure in patients with chronic atrial fibrillation. *J Am Soc Echocardiogr*. 2001;**14**:1080-1087

22. Nagueh SF, Kopelen HA, Quinones MA. Assessment of left ventricular filling pressures by Doppler in the presence of atrial fibrillation. *Circulation*. 1996;**94**:2138-2145

23. Okura H, Takada Y, Kubo T, Iwata K, Mizoguchi S, Taguchi H, *et al.* Tissue Doppler-derived index of left ventricular filling pressure, E/E', predicts survival of patients with non-valvular atrial fibrillation. *Heart*. 2006;**92**:1248-1252

24. Oyama R, Murata K, Tanaka N, Takaki A, Ueda K, Liu J, *et al.* Is the ratio of transmitral peak E-wave velocity to color flow propagation velocity useful for evaluating the severity of heart failure in atrial fibrillation? *Circ J*. 2004;**68**:1132-1138

25. Peltier M, Leborgne L, Zoubidi M, Slama M, Tribouilloy CM. Prognostic value of short-deceleration time of mitral inflow E velocity: implications in patients with atrial

fibrillation and left-ventricular systolic dysfunction. *Arch Cardiovasc Dis*. 2008;**101**:317-325

26. Punjani S, Wu WC, Cohen S, Sharma SC, Choudhary G. Echocardiographic indices of diastolic function relate to functional capacity and quality of life in ambulatory men with atrial fibrillation. *J Am Soc Echocardiogr*. 2011;**24**:533-540 e533

27. Senechal M, O'Connor K, Deblois J, Magne J, Dumesnil JG, Pibarot P*, et al.* A simple Doppler echocardiography method to evaluate pulmonary capillary wedge pressure in patients with atrial fibrillation. *Echocardiography*. 2008;**25**:57-63

28. Schneider F, Martin DT, Schick EC, Gaasch WH. Interval-dependent changes in left ventricular contractile state in lone atrial fibrillation and in atrial fibrillation associated with coronary artery disease. *Am J Cardiol*. 1997;**80**:586-590

29. Shahgaldi K, Manouras A, Abrahamsson A, Gudmundsson P, Brodin LA, Winter R. Three-dimensional echocardiography using single-heartbeat modality decreases variability in measuring left ventricular volumes and function in comparison to four-beat technique in atrial fibrillation. *Cardiovasc Ultrasound*. 2010;**8**:45

30. Sohn DW, Song JM, Zo JH, Chai IH, Kim HS, Chun HG*, et al.* Mitral annulus velocity in the evaluation of left ventricular diastolic function in atrial fibrillation. *J Am Soc Echocardiogr*. 1999;**12**:927-931

31. Su HM, Lin TH, Hsu PC, Chu CY, Lee WH, Lee CS*, et al.* Myocardial performance index derived from preejection period: a novel and feasible parameter in evaluation of cardiac performance in patients with permanent atrial fibrillation. *Echocardiography*. 2011;**28**:1081-1087

32. Su HM, Lin TH, Hsu PC, Lee WH, Chu CY, Lee CS*, et al.* Global left ventricular longitudinal systolic strain as a major predictor of cardiovascular events in patients with atrial fibrillation. *Heart*. 2013;**99**:1588-1596

33. Thavendiranathan P, Liu S, Verhaert D, Calleja A, Nitinunu A, Van Houten T*, et al.* Feasibility, accuracy, and reproducibility of real-time full-volume 3D transthoracic echocardiography to measure LV volumes and systolic function: a fully automated endocardial contouring algorithm in sinus rhythm and atrial fibrillation. *JACC Cardiovasc Imaging*. 2012;**5**:239-251

34. Temporelli PL, Scapellato F, Corra U, Eleuteri E, Imparato A, Giannuzzi P. Estimation of pulmonary wedge pressure by transmitral Doppler in patients with chronic heart failure and atrial fibrillation. *Am J Cardiol*. 1999;**83**:724-727

35. Traversi E, Cobelli F, Pozzoli M. Doppler echocardiography reliably predicts pulmonary artery wedge pressure in patients with chronic heart failure even when atrial fibrillation is present. *Eur J Heart Fail*. 2001;**3**:173-181

36. Wada Y, Murata K, Tanaka T, Nose Y, Kihara C, Uchida K*, et al.* Simultaneous Doppler tracing of transmitral inflow and mitral annular velocity as an estimate of elevated left ventricular filling pressure in patients with atrial fibrillation. *Circ J*. 2012;**76**:675-681

37. Wang CL, Lin KH, Luqman N, Chu PH, Hsu LA, Kuo CT. Simultaneous biplane single-

beat assessment of left ventricular systolic function in patients with atrial fibrillation. *Am J Cardiol*. 2004;**94**:942-944

38. Wang CL, Lin KH, Luqman N, Ho WJ, Hsu LA, Chu PH, *et al.* Refinement on single-beat determination of left ventricular systolic function in patients with atrial fibrillation. *J Am Soc Echocardiogr*. 2005;**18**:913-918

39. Wang CL, Ho WJ, Luqman N, Hsu LA, Kuo CT. Biplane assessment of left ventricular function during atrial fibrillation at beats with equal subsequent cycles. *Int J Cardiol*. 2006;**113**:54-60

40. Ko HS, Kim CJ, Ryu WS. New parameters for left ventricular function in atrial fibrillation: based on the relationship between RR interval and performance. *J Korean Med Sci*. 2005;**20**:20-25

41. Simpson E, Stevenson M, Scope A, Poku E, Minton J, Evans P. Echocardiography in newly diagnosed atrial fibrillation patients: a systematic review and economic evaluation. *Health Technol Assess*. 2013;**17**:1-263, v-vi

42. Kotecha D, Chudasama R, Lane DA, Kirchhof P, Lip GY. Atrial fibrillation and heart failure due to reduced versus preserved ejection fraction: A systematic review and meta-analysis of death and adverse outcomes. *Int J Cardiol*. 2016;**203**:660-666

43. Kotecha D, Holmes J, Krum H, Altman DG, Manzano L, Cleland JG, *et al.* Efficacy of beta blockers in patients with heart failure plus atrial fibrillation: an individual-patient data meta-analysis. *Lancet*. 2014;**384**:2235-2243

44. Ziff OJ, Lane DA, Samra M, Griffith M, Kirchhof P, Lip GY, *et al.* Safety and efficacy of digoxin: systematic review and meta-analysis of observational and controlled trial data. *BMJ*. 2015;**351**:h4451

45. Tighe DA, Vinch CS, Hill JC, Meyer TE, Goldberg RJ, Aurigemma GP. Influence of age on assessment of diastolic function by Doppler tissue imaging. *Am J Cardiol*. 2003;**91**:254-257

46. Ommen SR, Nishimura RA, Appleton CP, Miller FA, Oh JK, Redfield MM, *et al.* Clinical utility of Doppler echocardiography and tissue Doppler imaging in the estimation of left ventricular filling pressures: A comparative simultaneous Doppler-catheterization study. *Circulation*. 2000;**102**:1788-1794

47. Kim SA, Park WJ, Kim HJ, Jo SH, Kim HS, Han SJ, *et al.* Determinants of preserved diastolic function at the lateral annulus in patients with isolated diastolic dysfunction. *Eur Heart J Cardiovasc Imaging*. 2013;**14**:443-448

48. Nagueh SF, Mikati I, Kopelen HA, Middleton KJ, Quinones MA, Zoghbi WA. Doppler estimation of left ventricular filling pressure in sinus tachycardia. A new application of tissue doppler imaging. *Circulation*. 1998;**98**:1644-1650

49. Ritzema JL, Richards AM, Crozier IG, Frampton CF, Melton IC, Doughty RN, *et al.* Serial Doppler echocardiography and tissue Doppler imaging in the detection of elevated directly measured left atrial pressure in ambulant subjects with chronic heart failure. *JACC Cardiovasc Imaging*. 2011;**4**:927-934

50. Sharifov OF, Schiros CG, Aban I, Denney TS, Gupta H. Diagnostic Accuracy of Tissue Doppler Index E/e' for Evaluating Left Ventricular Filling Pressure and Diastolic Dysfunction/Heart Failure With Preserved Ejection Fraction: A Systematic Review and Meta-Analysis. *J Am Heart Assoc*. 2016;**5**

51. Sengelov M, Jorgensen PG, Jensen JS, Bruun NE, Olsen FJ, Fritz-Hansen T, *et al.* Global Longitudinal Strain Is a Superior Predictor of All-Cause Mortality in Heart Failure With Reduced Ejection Fraction. *JACC Cardiovasc Imaging*. 2015;**8**:1351-1359

52. Institute of Cardiovascular Sciences; University of Birmingham, Chief Investigator Kotecha D. Rate Control Therapy Evaluation in Permanent Atrial Fibrillation (RATE-AF). *National Library of Medicine (US)*. 2015; **NLM Identifier: NCT02391337 [Accessed 20-Nov-2015]**: Available from: https://clinicaltrials.gov/ct2/show/NCT02391337

Conflicts

All authors have completed the ICMJE conflict of interest statement and report no conflicts. DK discloses non-financial support from Daiitchi Sankyo, research grants from Menarini and lecture fees from AtriCure, all outside the submitted work; and Lead for the Beta-blockers in Heart Failure Collaborative Group (BB-meta-HF) and the RAte control Therapy Evaluation in Atrial Fibrillation (RATE-AF) trial. MM, ES and RS have no disclosures to report. BAP discloses research support and honoraria from GE Healthcare and Hitachi-Aloka, all outside the submitted work.

Funding

DK is supported by a National Institute of Health Research (NIHR) Career Development Fellowship (CDF-2015-08-074). The opinions expressed in this paper are those of the authors and do not represent the NIHR or the UK Department of Health.

Statement

The manuscript has neither been published (except in the form of abstract or thesis) nor is currently under consideration for publication by any other journal. All named authors have seen and approved the final version of the manuscript.

Table 1: Summary of included studies

Study	Number with AF	Population	Relevant topic(s)	Aims and methods	Main findings related to AF
**Belenkie, 1979** [8]	11	Patients with sinus rhythm and AF, excluding technically inadequate echocardiograms.	Acquisition.	Association of end diastolic dimension and cycle length with M-mode parameters of LV systolic function.	Preload and cycle length correlated with LVEF. Patients with AF had higher correlation of RR interval with LVEF than patients with sinus rhythm.
**Benyounes, 2015** [9]	17	Consecutive patients including those with AF, but no important variability in heart rhythm.	Systolic validity.	Internal validation of strain measurement against LVEF.	High correlation of strain and LVEF in AF patients, and probably similar to sinus rhythm.
**Chirillo, 1997** [10]	35	AF for >3 months without mitral stenosis, undergoing catheterization on intensive care or electively.	Diastolic validity.	Correlation of invasive PCWP with mitral and PV flow velocities and derivation of non-invasive algorithm.	Diastolic PV flow better than mitral indices for estimating PCWP in AF.
**Diwan, 2005** [11]	13	Consecutive patients with mitral valve disease undergoing catheterization.	Diastolic validity.	Correlation of invasive PCWP with Doppler indices of diastolic function.	The ratio of IVRT to the time period between E and e' highly correlated with PCWP in AF, similar to sinus rhythm.
**Dubrey, 1997** [12]	21	Selected AF patients with irregularity of rate on electrocardiogram.	Systolic reproducibility and acquisition.	Variability in LV outflow tract Doppler in AF compared to sinus rhythm.	13 beats required in AF to achieve variability <2%, compared to 4 beats in sinus rhythm.
**Galderisi, 1992** [13]	12	Patients randomly selected from the Framingham cohort with heart rate <100 beats/min and technically adequate Doppler.	Diastolic reproducibility.	Reproducibility of Doppler indices of diastolic function in sinus rhythm and AF.	Variability similar in AF and sinus rhythm. Reproducibility highest for peak velocity and area under the curve rather than slope measures.
**Kerr, 1998** [14]	38	Consecutive non-valvular AF patients with good quality echocardiography.	Acquisition.	Impact of heart rate cycle length variability on LV outflow tract Doppler.	Variability in stroke volume increased at higher heart rates in AF.
**Kusunose, 2009** [15]	56	Non-valvular AF patients with preserved systolic function (n=21 with simultaneous catheterization).	Diastolic validity and reproducibility.	Validation of single-beat E/e' recorded by synchronous dual Doppler.	Single-beat lateral E/e' a reliable indicator of elevated PCWP and plasma BNP in AF patients.
**Kusunose, 2012** [16]	25	Prospective assessment of non-valvular AF patients referred for catheterization.	Systolic reproducibility and acquisition.	Validation of an index-beat assessment versus 10-second average for myocardial strain and strain rate.	A single index-beat (with similar preceding and pre-preceding RR intervals) was accurate compared to averaged mean values.
**Lee, 2005** [17]	73	Non-valvular chronic AF with heart rate ≤100 beats/min and clinically stable.	Systolic and diastolic validity.	Correlation of clinical and echocardiographic parameters with maximum symptom-limited treadmill.	E/e' significantly related to exercise capacity in AF, unlike other echocardiographic parameters.

Study	Number with AF	Population	Relevant topic(s)	Aims and methods	Main findings related to AF
**Lee, 2008** [18]	330	Multicentre consecutive patients with persistent AF, LVEF >40% and heart rate ≤100 beats/min.	Systolic and diastolic validity.	Identification of echocardiographic risk factors for retrospective ischemic stroke.	E/e' significantly associated with prior stroke in AF patients with LVEF >40%.
**Lee, 2012** [19]	98	Prospective study of persistent or permanent AF patients with heart rate ≤105 beats/min.	Systolic reproducibility and acquisition.	Validation of index-beat measurement of LV peak longitudinal systolic strain.	A single index-beat was accurate compared to averaging multiple cardiac cycles.
**Li, 2010** [20]	49	Non-valvular AF patients with preserved ejection fraction undergoing catheterization.	Diastolic validity and reproducibility.	Correlation of single-beat E/e' with invasive PCWP.	Stronger association between E/e' and PCWP using a single-beat, dual Doppler method.
**Matsukida, 2001** [21]	32	Chronic AF patients undergoing catheterization.	Diastolic validity and reproducibility.	Correlation of diastolic indices with invasive PCWP.	PV flow and deceleration time independently associated with PCWP.
**Nageuh, 1996** [22]	60	Non-valvular AF patients (majority intensive care or surgical patients).	Diastolic validity and reproducibility.	Correlation of diastolic indices with invasive PCWP in training and test groups.	Diastolic indices (e.g. IVRT) highly correlated with PCWP, particularly in AF patients with LVEF <45%.
**Okura, 2006** [23]	230	Retrospective analysis of consecutive non-valvular AF patients.	Diastolic validity and reproducibility.	Assessment of E/e' at a cut-point of 15 as a predictor of mortality over a follow-up period of 245 (± 200) days.	AF patients with E/e' >15 have higher mortality, independent of clinical factors.
**Oyama, 2004** [24]	68	Non-valvular chronic AF patients.	Diastolic validity and reproducibility.	Correlation of E/Vp using single-beat dual Doppler with plasma BNP concentration and invasive PCWP.	E/Vp associated with both BNP and PCWP.
**Peltier, 2008** [25]	40	Prospective assessment of patients with non-valvular AF >1 month and LVEF <40%, hospitalized for heart failure.	Diastolic validity and reproducibility.	Correlation of E/e' with functional capacity and quality of life.	Deceleration time <150ms was independently associated with mortality in both AF and sinus rhythm.
**Punjani, 2011** [26]	48	Retrospective analysis of persistent or permanent AF with LVEF ≥50% and heart rate ≤100 beats/min.	Diastolic validity and reproducibility.	Determine relationship between diastolic parameters and functional capacity/quality of life, when measured on two different occasions 1 week apart.	E/e' independently associated with walk distance and quality of life in patients with AF and preserved LVEF.
**Schneider, 1997** [28]	18	Chronic AF patients during routine echocardiography.	Acquisition.	Test hypothesis that LV systolic function is affected by pre-preceding cycle length.	Pre-preceding RR interval has an important effect on LV peak ejection velocity.
**Senechal, 2008** [27]	24	Consecutive early post-operative patients with predominantly paroxysmal AF and no mitral prosthesis.	Diastolic validity, diastolic reproducibility and acquisition.	E/e' for estimating invasive PCWP with comparison of 10-beat average and one cycle with the longest R-R interval.	E/e' with a single cardiac cycle had similar correlation with PCWP as averaged measures.

Study	Number with AF	Population	Relevant topic(s)	Aims and methods	Main findings related to AF
**Shahgaldi, 2010** [29]	23	Consecutive patients referred for echocardiography.	Systolic reproducibility.	Comparison of 1-beat and 4-beat 3D volumes and LVEF in patients with sinus rhythm and AF.	Lower variability in 3D full volume acquisition in AF patients using a 1-beat rather than 4-beat acquisition.
**Sohn, 1999** [30]	27	Non-valvular AF patients undergoing catheterization.	Diastolic validity.	Correlation of E/e' with invasive PCWP.	E/e' highly correlated with PCWP.
**Su, 2011** [31]	54	Consecutive patients with permanent AF and adequate echocardiographic images.	Systolic reproducibility.	Validation of pre-ejection period myocardial performance index with other indices of systolic and diastolic function in AF.	Pre-ejection period myocardial performance index is an indicator of global LV function in permanent AF.
**Su, 2013** [32]	196	Prospective assessment of consecutive patients with persistent AF and adequate images.	Systolic validity and reproducibility.	Ability of global longitudinal strain to predict cardiovascular events over follow-up of 21 (±10) months.	Global longitudinal strain improved prediction of adverse events beyond LVEF and tissue Doppler assessment.
**Temporelli, 1999** [34]	35	Patients with heart failure, LVEF <35%, AF >3 months and acceptable images.	Diastolic validity and reproducibility.	Correlation of diastolic indices with invasive PCWP.	Deceleration time was independently associated with PCWP in AF patients with severe LV dysfunction.
**Thavendiranthan, 2012** [33]	24	Subgroup of patients with AF referred for an echocardiogram (main study outcomes investigated patients with sinus rhythm).	Systolic validation.	Assessment of an automated edge contouring algorithm using real-time 3D acquisition, compared to conventional biplane Simpsons.	Automated 3D LVEF in AF patients correlated well with conventional LVEF analysis.
**Traversi, 2001** [35]	51	Patients with heart failure, LVEF <35%, AF >3 months and heart rate <90 beats/min, as part of a pre-transplant evaluation.	Diastolic validity and reproducibility.	Correlation of diastolic indices with invasive PCWP.	Mitral and PV flow indices correlate with PCWP in AF patients assessed for heart transplantation.
**Wada, 2012** [36]	45	Non-valvular chronic AF patients with normal right ventricular function.	Diastolic validity and reproducibility.	Correlation of single-beat dual Doppler with invasive PCWP.	The time and ratio between E and e' correlated with PCWP, similar to BNP.
**Wang, 2004** [37]	40	Consecutive patients with AF and adequate acoustic windows.	Acquisition.	Evaluation of LVEF and stroke volume according to preceding cycle lengths.	Positive relationship between preceding cycle length and stroke volume.
**Wang, 2005** [38]	100	Consecutive AF patients referred for echocardiogram with adequate acoustic windows.	Acquisition.	Evaluation of aortic time-velocity integral according to preceding cycle length and varying beat repeats.	Assessment improved with cycle lengths >500ms and 2 or 3 beats with similar RR interval.
**Wang, 2006** [39]	75	Patients with AF referred for echocardiography with adequate acoustic windows.	Systolic reproducibility and acquisition	Improvement of systolic function evaluation according to cycle lengths and number of repeated beats.	LVEF and stroke volume can be reliably obtained in AF by averaging two beats with similar preceding and pre-preceding RR intervals and cycle length >500ms.

3D = Three-dimensional; AF = atrial fibrillation; BNP = B-type natriuretic peptide; IVRT = isovolumic relaxation time; LV = left ventricular; LVEF = left ventricular ejection fraction; PCWP = pulmonary capillary wedge pressure; PV = pulmonary vein.

Table 2: Pooled characteristics

Characteristic	Range of reported means	Weighted average (standard deviation of means)	Number of studies / number of patients
Age	57 - 76 years	66.9 (4.5) years	31 / 1916
Women	0 – 52 %	33 (11) %	27 / 1835
Hypertension	17 - 85 %	53 (18) %	11 / 1235
Heart failure	0 - 100 %	48 (35) %	14 / 1473
LVEF	22 - 65 %	52.5 (9.7) %	25 / 1646
E/e’:			
Average	9 – 23	11.7 (2.7)	5 / 437
Septal	11 – 23	13.4 (4.7)	2 / 560
Lateral	8 – 14	10.3 (2.1)	5 / 196
Heart rate	63 - 107 beats/min	79.9 (6.3) beats/min	20 / 1223

Pooled baseline characteristics, weighted according to sample size. E/e’ = ratio of mitral peak E velocity and tissue Doppler early diastolic filling e’; LVEF = left ventricular ejection fraction.

Table 3: Reproducibility of systolic echocardiographic measures in AF

Parameter / Study	N	REPRODUCIBILITY<br>Intra-observer and inter-observer variability
**Simpson's LVEF:**<br>Wang, 2006 [39]	10	Single-beat intra 2.8%
**3-dimensional LVEF:**<br>Shahgaldi, 2010 [29]	23	4-beat intra 8.3%, inter 17.9%<br>Single beat intra 4.8%, inter 5.6%
**Peak longitudinal systolic strain:**<br>Lee, 2012 [19]	15	15-cycle average intra 2.4%, inter 2.7%<br>Single index beat intra 3.5%, inter 4.0%
**Global longitudinal strain*:**<br>Su, 2013 [32]	30	Intra 5.3%, inter 6.2%
**Myocardial performance index†:**<br>Su, 2011 [31]	54	Intra 5.2%, inter 7.3%

LVEF = left ventricular ejection fraction; * Using single index beat; † A marker of combined systolic and diastolic function calculated as the sum of pre-ejection time and isovolumic relaxation time as a ratio of ejection time.

Table 4: Validity and reproducibility of diastolic echocardiographic measures in AF

Parameter / Study	N	DIASTOLIC VALIDATION<br>Correlation with invasive pulmonary capillary wedge pressure (r)	DIASTOLIC REPRODUCIBILITY<br>Intra-observer and inter-observer mean differences (MD) ± standard deviation, coefficient of variation (CV), retest correlation (RC) or retest variability (RV)	Mean LVEF (SD) %
**Isovolumic relaxation time (IVRT):**				
Nagueh, 1996 [22]	30	-0.76‡	Intra MD 1.4±8.4ms, inter MD 4.5±9.0ms‖	45 (16)
Temporelli, 1999 [34]	35	-0.95‡	CV 1.9-2.4%¶	22 (5)
Traversi, 2001 [35]	51	-0.70‡	Intra MD 0.15±0.15, inter MD 0.25±1.64mmHg††	25 (7)
Diwan, 2005 [11]	13	-0.92†,§		54 (11)
Punjani, 2011 [26]	48		Intra RC 0.54	
**Mitral E wave deceleration time:**				
Galderisi, 1992 [13]	12		Intra RC 0.85-0.93, inter RC 0.76	
Nagueh, 1996 [22]	30	-0.42*	Intra MD 1.0±4.0ms; inter MD 5.4±7.8ms‖	45 (16)
Chirillo, 1997 [10]	35	-0.50†	CV "not statistically significant"	41 (13)
Sohn, 1999 [30]	27	no correlation		53 (11)
Temporelli, 1999 [34]	35	-0.70†	CV 1.9-2.4%¶	22 (5)
Matsukida, 2001 [21]	32	-0.65‡	Intra RV 5.1%, inter RV 5.6%¶	‡‡
Traversi, 2001 [35]	51	-0.60‡		25 (7)
Peltier, 2008 [25]	30		Intra RC 0.88, inter RC 0.84.	31 (8)
Senechal, 2008 [27]	24	no correlation	Intra RV 1.2-3.6%, inter RV 2.3-4.8%¶, **	46 (15)
Punjani, 2011 [26]	48		Intra RC 0.75	
**Ratio of mitral peak E velocity and tissue Doppler e' (E/e'):**				
Sohn, 1999 [30]	27	Septal 0.79‡		53 (11)
Okura, 2006 [23]	230		Septal intra RV 5.0%, inter RV 11.4%	56 (12)
Senechal, 2008 [27]	24	Lateral 0.47*, septal 0.46*	Intra RV 1.2-3.6%, inter RV 2.3-4.8%¶, **	46 (15)
Kusunose, 2009 [15]	21	Lateral 0.57†, single-beat lateral 0.74‡	Single-beat lateral intra RV 4.9%, inter RV 6.6%#	60 (6)
Li, 2010 [20]	49	Lateral 0.49‡, single-beat lateral 0.77‡	Single-beat lateral intra RV 6.7%, inter RV 7.9%	59 (8)
Punjani, 2011 [26]	48		Lateral intra RC 0.84, septal intra RC 0.86	
Wada, 2012 [36]	45	Average single-beat 0.57‡	Single-beat average intra RV 4.3%, inter RV 11.1%	52 (16)
**Ratio of mitral peak E velocity and velocity of diastolic flow propagation (E/Vp):**				
Nagueh, 1996 [22]	30	0.65‡	Intra MD 0.2±0.4ms, inter MD 0.13±0.40ms‖	45 (16)
Oyama, 2004 [24]	25	0.63†	Intra RV 5.1%, inter 5.3%	55 (15)
Punjani, 2011 [26]	48		Intra RC 0.79	
**Pulmonary venous flow diastolic wave deceleration time (PVd-DT):**				
Chirillo, 1997 [10]	35	-0.91‡	CV "not statistically significant"	41 (13)
Matsukida, 2001 [21]	32	-0.80‡	Intra RV 5.1%, inter RV 5.6%¶	‡‡

LVEF = left ventricular ejection fraction. * p≤0.05; † p≤0.01; ‡ p<0.001; § IVRT as a ratio to the difference between onset time of mitral E and annulus e' velocities; ‖ N=60 for reproducibility data; ¶ Combined reproducibility assessment for all Doppler variables; # N=10 for reproducibility data; ** N=6 for reproducibility data; †† N=40 for reproducibility data; based on a composite of IVRT, deceleration rate and systolic fraction; ‡‡ Fractional shortening 29% (SD 4%). Retest variability typically expressed as the mean percentage error.

Supplementary Table A: Risk of Bias assessment

Study	Selection of participants	Confounding variables	Measurement of exposure	Blinding of outcome	Incomplete outcome data	Selective reporting
Belenkie, 1979 [8]	High	High	High	High	Low	Low
Benyounes, 2015 [9]	High	High	Low	High	High	Unclear
Chirillo, 1997 [10]	Low	Low	Unclear	High	Low	Low
Diwan, 2005 [11]	Unclear	Unclear	Low	High	High	Unclear
Dubrey, 1997 [12]	Low	Low	High	High	Unclear	Unclear
Galderisi, 1992 [13]	Low	Low	Low	High	Low	Low
Kerr, 1998 [14]	Low	Low	Low	Low	Low	Unclear
Kusunose, 2009 [15]	Low	Low	Low	High	Low	Low
Kusunose, 2012 [16]	High	Low	Low	High	Low	Low
Lee, 2005 [17]	High	Low	High	High	High	Unclear
Lee, 2008 [18]	Low	Unclear	High	High	Unclear	Unclear
Lee, 2012 [19]	High	Low	High	High	Unclear	Low
Li, 2010 [20]	High	Low	Low	High	Unclear	Unclear
Matsukida, 2001 [21]	Low	Low	Low	Unclear	Unclear	Unclear
Nageuh, 1996 [22]	Unclear	High	Unclear	High	Low	Low
Okura, 2006 [23]	High	Low	Low	High	Unclear	Unclear
Oyama, 2004 [24]	Unclear	High	High	High	High	Unclear
Peltier, 2008 [25]	High	Low	Unclear	Low	Low	Unclear
Punjani, 2011 [26]	High	High	High	Low	Low	Low
Schneider, 1997 [28]	High	High	High	High	Low	High
Senechal, 2008 [27]	High	Unclear	High	High	Low	Unclear
Shahgaldi, 2010 [29]	Low	Low	Low	Low	Unclear	Low
Sohn, 1999 [30]	High	High	High	High	Unclear	Unclear
Su, 2011 [31]	High	Low	High	High	Low	Unclear
Su, 2013 [32]	Low	Low	Low	Low	Low	Low
Temporelli, 1999 [34]	High	Low	High	High	High	High
Thavendiranthan, 2012 [33]	High	Low	Low	Low	Unclear	Low
Traversi, 2001 [35]	High	High	Low	High	Unclear	Unclear
Wada, 2012 [36]	High	Low	High	High	Unclear	Unclear
Wang, 2004 [37]	High	Low	High	High	Unclear	Unclear
Wang, 2005 [38]	High	Low	High	High	Unclear	Unclear
Wang, 2006 [39]	High	Low	High	High	Unclear	Unclear

Figure legends

Figure 1: Systematic review flowchart

AF = atrial fibrillation.

Figure 2: Example of optimal acquisition (index beat method)

In order to achieve the most valid and reproducible measurement in atrial fibrillation, parameters should be acquired where the two preceding cardiac cycles have similar RR-intervals and preferably where the equivalent heart rate is <100 beats/minute (panel A). This method can also be applied to assessment of function; averaging individual index beats is preferable to averaging across sequential cardiac cycles (panel B).

Figure 3: Summary of findings for echocardiography in AF

AF = atrial fibrillation; E/e' = ratio of mitral peak E velocity and tissue Doppler early diastolic filling e'; E/Vp = ratio of mitral peak E velocity and the velocity of diastolic flow propagation; IVRT = isovolumic relaxation time; PCWP = pulmonary capillary wedge pressure; PVd-DT = pulmonary venous diastolic flow deceleration time.

Figure 1

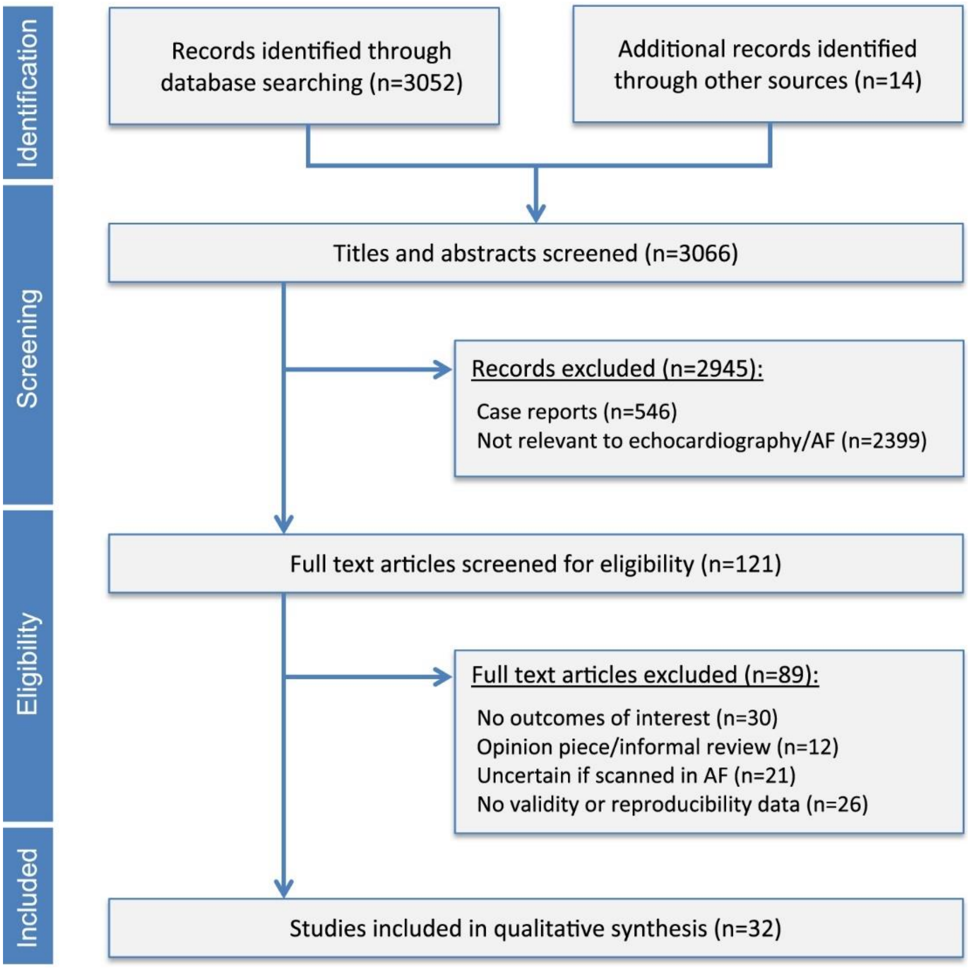

Identification
Screening
Eligibility
Included
Records identified through database searching (n=3052)
Additional records identified through other sources (n=14)
Titles and abstracts screened (n=3066)
Records excluded (n=2945):
Case reports (n=546)
Not relevant to echocardiography/AF (n=2399)
Full text articles screened for eligibility (n=121)
Full text articles excluded (n=89):
No outcomes of interest (n=30)
Opinion piece/informal review (n=12)
Uncertain if scanned in AF (n=21)
No validity or reproducibility data (n=26)
Studies included in qualitative synthesis (n=32)

Figure 2

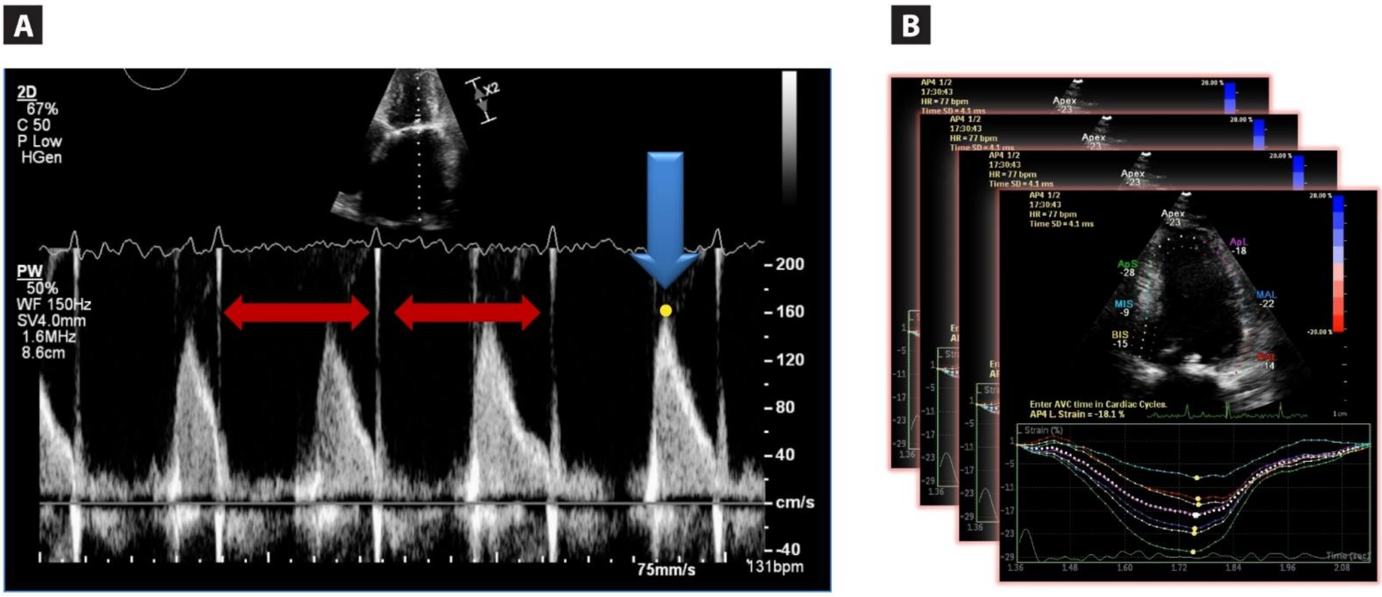
A
B
2D
67%
C 50
P Low
HGen
PW
50%
WF 150Hz
SV4.0mm
1.6MHz
8.6cm
200
160
120
80
40
cm/s
-40
75mm/s
131bpm
Apex
ApL
ApS
MIS
MAL
BIS
Enter AVC time in Cardiac Cycles.
AP4 L. Strain = -18.1 %
Strain (%)
Time (sec)

Figure 3

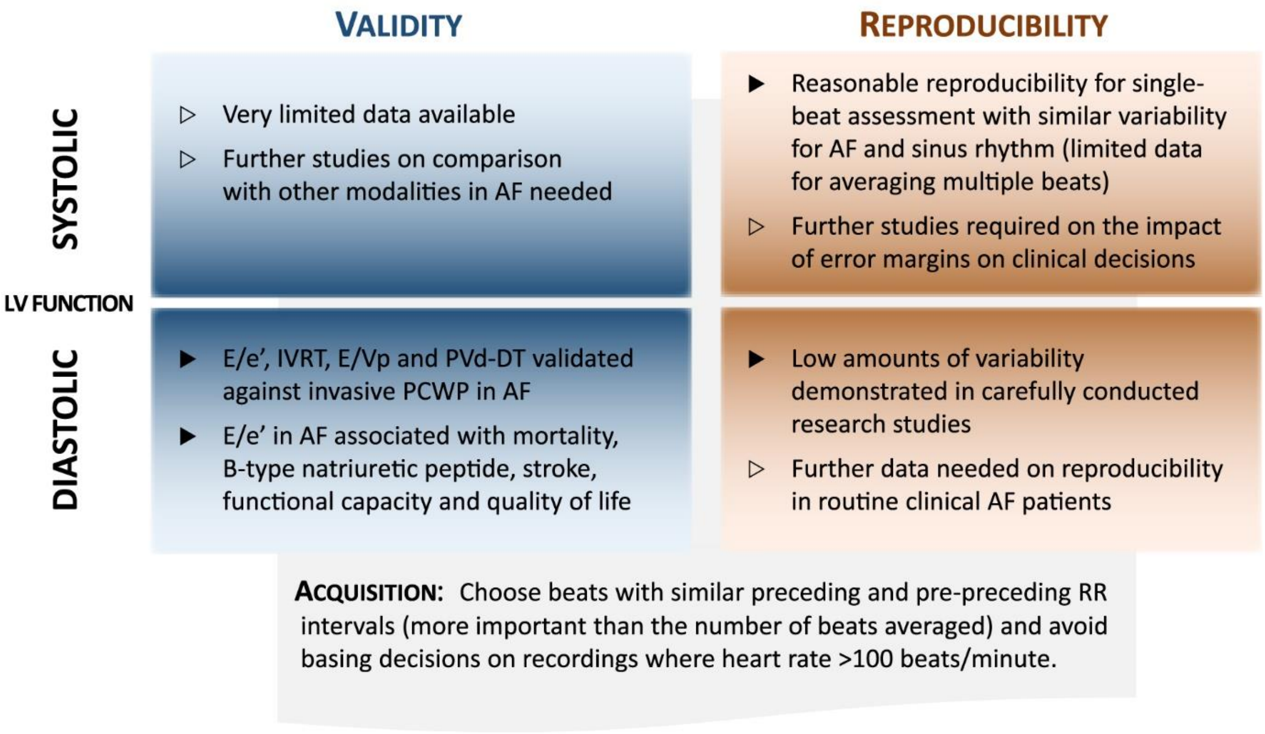
VALIDITY
REPRODUCIBILITY
SYSTOLIC
LV FUNCTION
DIASTOLIC
▷ Very limited data available
▷ Further studies on comparison with other modalities in AF needed
▶ Reasonable reproducibility for single-beat assessment with similar variability for AF and sinus rhythm (limited data for averaging multiple beats)
▷ Further studies required on the impact of error margins on clinical decisions
▶ E/e', IVRT, E/Vp and PVd-DT validated against invasive PCWP in AF
▶ E/e' in AF associated with mortality, B-type natriuretic peptide, stroke, functional capacity and quality of life
▶ Low amounts of variability demonstrated in carefully conducted research studies
▷ Further data needed on reproducibility in routine clinical AF patients
ACQUISITION: Choose beats with similar preceding and pre-preceding RR intervals (more important than the number of beats averaged) and avoid basing decisions on recordings where heart rate >100 beats/minute.

Manuscript CLEAN VERSION

Click here to download Manuscript (incl.refs, Acknowledgments, Funding, Ethics, etc follow guidelines)

Title: Is echocardiography valid and reproducible in patients with atrial fibrillation? A systematic review

Brief Title: Echocardiography in atrial fibrillation

Authors: Dipak Kotecha[1,2,3*], Mohamed Mohamed[4], Eduard Shantsila[1], Bogdan A Popescu[5], Richard P Steeds[1,3]

From the (1) University of Birmingham Institute of Cardiovascular Sciences, Birmingham, UK; (2) Centre of Cardiovascular Research and Education in Therapeutics, Monash University, Melbourne, Australia; (3) University Hospitals Birmingham NHS Trust, Birmingham, UK; (4) East Kent Hospitals University NHS Trust, Ashford, UK; and (5) University of Medicine and Pharmacy 'Carol Davila' - Euroecolab, and Institute of Cardiovascular Diseases 'Prof. C. C. Iliescu' Bucharest, Romania.

*Address for correspondence:

Dr Dipak Kotecha MBChB PhD MRCP FESC FHEA

University of Birmingham Institute of Cardiovascular Sciences,

The Medical School, Vincent Drive, Edgbaston, Birmingham, B15 2TT, United Kingdom.

Email: d.kotecha@bham.ac.uk

Tel: +44 121 507 5080 Fax: +44 121 554 4083

Word count (abstract): 249

Word count (text, excl. references): 3387

Key Words: Atrial fibrillation; Echocardiography; Reproducibility; Ejection fraction; Heart failure; Diastolic.

Abstract

Aims: Echocardiography is vital in the routine assessment and management of atrial fibrillation (AF). We performed a systematic review of the validity and reproducibility of echocardiographic left ventricular systolic and diastolic function in AF, and optimal acquisition methods.

Methods: Online databases were searched for studies in patients with AF at the time of echocardiography (1960 to August 2015), prospectively registered with PROSPERO (CRD42015025297).

Results: The systematic review included 32 studies from 3,066 search results (1,968 patients with AF). Average age was 67 years, 33% were women, mean LVEF 53% (±10%) and average E/e' 11.7 (±2.7). Data on the validity and reproducibility of systolic indices were extremely limited. In contrast, diastolic parameters demonstrated correlation with invasive filling pressure and adequate reproducibility: E/e' (n=444) r= 0.47 to 0.79; IVRT (n=177) r= -0.70 to -0.95; E/Vp (n=55) r= 0.63 and 0.65; pulmonary vein diastolic flow (n=67) r= -0.80 and -0.91. Elevated E/e' (>15) was associated with functional capacity, quality of life and impaired prognosis. For optimal acquisition in AF patients, cardiac cycles with controlled heart rate (<100 beats/minute) and similar preceding and pre-preceding RR intervals are required. Cardiac cycle length and equivalence were more important that the number of beats averaged.

Conclusions: With careful selection of appropriate cardiac cycles, echocardiography is a valid tool to identify diastolic dysfunction in AF, and E/e' is an independent marker of clinical status and adverse prognosis. However, data on systolic function was extremely limited and requires further prospective study and assessment of variability in clinical practice.

Condensed abstract (50 words)

Echocardiography is recommended in all AF patients, but validity and reproducibility is unclear. In this systematic review, we identified a lack of data on systolic function, whereas diastolic parameters have been validated against invasive pressure with adequate reproducibility. Optimal acquisition requires the two preceding cardiac cycles to have similar RR-intervals.

What's New? (113 words)

1. The new 2016 ESC Guidelines on AF recommend echocardiography in all AF patients to guide management (I C).
2. In this systematic review, data on the validity and reproducibility of systolic indices in AF patients were extremely limited; the best measure of systolic function and acquisition method in AF are priorities for future research.
3. Diastolic parameters in AF have been validated against invasive filling pressure with adequate reproducibility. Elevated E/e' (>15) is also associated with functional capacity, quality of life and impaired prognosis.
4. Measurement of systolic and diastolic function in AF is optimised when the two preceding cardiac cycles have similar RR-intervals and the heart rate is controlled (<100 beats/minute).

Introduction

Atrial fibrillation (AF) is an increasingly common heart rhythm disturbance that leads to frequent hospital admissions, heart failure, stroke, and higher mortality.[1] There is a close relationship between AF and heart failure, with numerous risk factors common to both conditions and shared pathophysiology in patients with both reduced [2] and preserved [3] left ventricular ejection fraction (LVEF). Depending on the type of AF, the rate of prevalent heart failure is between 33% and 56% [4]; hence clinicians treating patients with AF need reliable information on both systolic and diastolic left ventricular (LV) function. Echocardiography is the primary tool used in clinical practice and provides vital guidance to determine appropriate use of anticoagulation, rate-control therapy and rhythm-control strategies, as well as important information on co-existing or precipitating pathology and prognostic data [5]. All of these important clinical decisions require echocardiographic measures that are valid and reproducible, regardless of cardiac rhythm.

The loss of synchronized atrial contraction and altered left atrial pressure is likely to affect the reproducibility of echocardiographic measurements in AF. Factors that have been implicated include the ratio of preceding to pre-preceding cycle length and heart rate during image acquisition. Both of these influence the volume of ejection and consequently the results of the most commonly-used measurements of LV function, particularly where these are taken over a number of cardiac cycles. Joint guidelines published by the American Society of Echocardiography and the European Association of Cardiovascular Imaging suggest a minimum of five beats in AF patients, although this is based on consensus opinion [6]. For diastolic function, the British Society of Echocardiography recommends averaging over 5-10 beats during cycle lengths equivalent to a heart rate between 60-80 beats/minute.

We performed a systematic and focused review of published literature on the use of echocardiography for determination of systolic and diastolic LV function in patients with AF. Our main objectives were to assess the validity of echocardiographic measures whilst in AF, both against other modalities and clinical outcomes, and the reproducibility of these parameters. A further objective was to appraise the acquisition of images. This includes the optimal number of repeated measurements and cardiac cycle lengths that would reduce variability of systolic and diastolic evaluation and allow confidence in the echocardiographic diagnosis of systolic or diastolic dysfunction in AF.

Methods

Eligibility criteria & search strategy

All studies reporting validity or reproducibility data on LV systolic or diastolic function in AF patients were examined. There was no restriction on study design, however only adult populations with AF at the time of echocardiography were considered. Exclusion criteria included case reports, animal studies and studies that were only published in abstract form or in a language other than English. All editorials, commentaries and informal reviews of other literature were also excluded, as were studies only assessing left atrial size or function. An online search was performed of PubMed and the Cochrane library (inception to December 2014, and then extended to August 2015), including the broad terms "atrial fibrillation" and "echocardiography" using MESH headings and title/abstract searches, including syntax variations. We also conducted manual screening of relevant reviews and reference lists. The systematic review was reported according to the Preferred Reporting Items for Systematic reviews and Meta-Analyses (PRISMA) guidelines and prospectively registered with the PROSPERO database of systematic reviews (http://www.crd.york.ac.uk/PROSPERO/display_record.asp?ID=CRD42015025297).

Outcomes

The primary outcomes of interest were echocardiographic measures of LV systolic and diastolic function. For systolic function, these included LVEF using biplane Simpson's method or 3D volume assessment and measurement of strain (peak longitudinal systolic strain [PLSS] and global longitudinal strain [GLS]). For diastolic function, we included assessment of isovolumic relaxation time (IVRT), mitral E-wave deceleration time, the ratio of mitral peak E velocity to tissue Doppler early diastolic velocity e' (E/e'), pulmonary venous (PV) flow diastolic

deceleration time (PVd-DT), and the ratio of mitral peak E velocity to the velocity of diastolic flow propagation measured with colour Doppler M-mode (E/Vp). For all parameters, we extracted data on validity against other modalities (for example, pulmonary capillary wedge pressure [PCWP] on right heart catheterization) and estimates of intra and inter-operator reproducibility. We also noted the method by which studies collected data, including the number of repeated measures and cardiac cycle lengths. A secondary outcome was to record average values of echocardiographic measures in AF, for comparison with published norms in patients with sinus rhythm.

Data collection and quality assessment

Data on validity (against other modalities and any relevant clinical associations) and reproducibility (both intra- and inter-observer variability) were extracted by three investigators independently (MM, ES and DK), and tabulated in a standardized data-extraction form. Study quality was assessed using the Risk of Bias Assessment Tool for Non-randomized Studies (RoBANS), which addresses selection bias, exposure measurement, blinding, the completeness of outcome data and selectivity of reporting [7]. Risk of bias was assessed by two investigators independently (MM and ES) and discrepancies resolved by group discussion and additional adjudication (DK).

Data synthesis and statistical analysis

Baseline demographics were pooled from all studies providing suitable data (including variance where applicable), and are summarized as a weighted mean according to sample size. Outcomes were synthesized qualitatively. Meta-analysis of comparative data between AF and sinus rhythm was not possible due to the limited studies available and a lack of published data on the variance of outcome measures. Analyses were performed on Stata Version 14.1 (StataCorp LP,

Texas).

Results

The search strategy identified a total of 3,066 records of which 2,945 were excluded, primarily due to lack of relevance to echocardiography in AF, and a further 89 excluded after full text review (**Figure 1**). Thirty-two observational studies were included in the final review [8-39], the majority of which were single-centre studies. **Table 1** highlights the populations examined and the key findings relating to patients with AF. There was marked heterogeneity in the type of AF (paroxysmal, persistent or permanent), heart failure status, LVEF and clinical demographics. The weighted-average age was 66.9 years and a third were women (**Table 2**). Overall, studies recorded a mean LVEF of 52.5% and average E/e' of 11.7 in AF. Heart rate was usually below 80 beats/minute, with a minority of studies excluding patients above a specific heart rate target (typically >100 beats/minute). Many studies excluded patients with AF due to valvular heart disease. Only four studies enrolled 100 or more patients, and there were frequent references to selecting participants with adequate quality echocardiographic images. As a result, the risk of bias for selection and blinding were universally high, although in other domains, the risk of bias was more variable (**Supplementary Table A**).

Systolic function: Validity and reproducibility

Data for the validity of systolic function indices in patients with AF were extremely limited. We found no external validation studies (for example, comparing results with other modalities such as cardiac magnetic resonance or nuclear imaging). There were however examples of within-study or internal validation (such as correlation of new 3D techniques with conventional biplane Simpsons, or strain with LVEF) [9, 33]. With respect to clinical outcomes, one study showed that

LV systolic parameters were unrelated to exercise capacity in 73 stable AF patients [17]. However, in a study of 196 patients with persistent AF, baseline GLS was independently associated with a composite of cardiovascular death, non-fatal stroke and heart failure hospitalization after 21 (±10) months follow-up [32]. This relationship persisted in multivariate analysis (hazard ratio 1.12, 95% CI 1.02-1.23, p=0.014), whereas LVEF and other measures of systolic function were not independently significant. The optimal, post-hoc defined GLS cut-off for predicting event-free survival was -12.5%, and this incrementally added to clinical predictors of adverse outcome [32].

Reproducibility of systolic function indices are summarized in **Table 3**. A wide array of study and acquisition methods made data synthesis unfeasible, however reproducibility was reasonable in AF patients using single-beat methods [19, 29, 31, 32, 39]. One study examining AF patients with irregularity on their electrocardiogram found that to achieve similar variability for cardiac output in AF as with sinus rhythm, three times the number of beats were required (13 versus 4 beats, respectively) [12]. In contrast, although there was higher inter-observer variability for 3D-LVEF using conventional 4-beat acquisition in AF compared to sinus rhythm (17.9% versus 3.9%, respectively), when using single-beat acquisition, reproducibility was similar regardless of heart rhythm (5.6% in AF, versus 4.5% in sinus rhythm) [29].

Diastolic function: Validity and reproducibility

Considerably more data were available for the use of diastolic parameters in AF (**Table 4**). Twenty studies provided correlations with invasive PCWP on right heart catheterization for a range of diastolic indices. IVRT was assessed in 4 studies (n=177) and inverse correlations with PCWP were all highly statistically significant, ranging from -0.70 to -0.95 [11, 22, 34, 35]. Seven studies examined mitral deceleration time (n=324), of which 2 found no correlation with PCWP

[27, 30] and 5 identified moderate inverse correlation [10, 21, 22, 34, 35]. All 5 studies of E/e’ (n=444) showed significant association with PCWP, ranging from 0.47 to 0.79, and including e’ derived from both septal and lateral positions [15, 20, 27, 30, 36]. Using a dual Doppler method, the combination of E/e’ and the time between E and e’ (cut-points at >14.6 and >34ms respectively), improved the sensitivity and specificity for predicting elevated PCWP versus either alone [36]. Compared to those in sinus rhythm, AF patients demonstrated a similar correlation with PCWP for the ratio of IVRT to time between E and e’ in patients with mitral valve disease [11]. E/Vp and the deceleration time of PV diastolic flow were each assessed in 2 studies (n=55 and n=67, respectively) and both parameters showed a high degree of correlation with PCWP [10, 21, 22, 24]. Diastolic PV flow was better than mitral indices for estimating PCWP in one study of 35 AF patients [10].

In regard to clinical outcomes, a retrospective analysis of 230 AF patients identified that septal E/e’ >15 was independently associated with mortality during follow-up of 245 (± 200) days, both in patients with impaired and preserved LVEF [23]. Deceleration time <150 ms was associated with mortality during follow-up of 25 (± 11) months in AF patients with LVEF <40% who had been hospitalized for heart failure, with a similar impact in AF patients (n=40) as those with sinus rhythm (n=100) [25]. Diastolic indices, including E/e’ and E/Vp, have also been shown to correlate with B-type natriuretic peptide (BNP), a biomarker strongly associated with adverse prognosis [15, 24]. E/e’ was the only echocardiographic variable of LV function related to exercise capacity in 73 patients with AF (multivariate adjusted coefficient β= -0.12; p= 0.032) [17]. The same group also showed in one of the only multicentre studies that septal E/e’ was associated with prior ischaemic stroke in 330 AF patients with LVEF >40% (adjusted odds ratio 1.21, 95% CI 1.08-1.37; p=0.002), unlike clinical and echocardiographic parameters such and age, BNP, or LVEF [18]. E/e’ also correlates with 6-minute walk distance and quality of life, as seen in a

retrospective study of 48 patients with AF and preserved LVEF [26].

Reproducibly of diastolic indices is summarized in **Table 4**, with intra- and inter-observer mean differences, coefficients of variation and test-retest variability reasonable in the majority of the 23 studies (n=997) [10, 13, 15, 20-27, 34-36]. Of note, E/e' was shown to be reliable when measured one week apart (correlation coefficient 0.87, p<0.05) [26], and the variability of diastolic indices was similar in AF and sinus rhythm in a small cohort of patients from the Framingham study [13].

Acquisition: Cycle length and cycle repeats

The irregular RR interval in AF has led to concern about the reliability of both systolic and diastolic measures, and there is clinical uncertainty about the number of repeated measures required and optimal cycle length. Historical data have shown that the RR interval affects LVEF in AF patients, more so than in sinus rhythm [8]. More recent studies have confirmed that the cycle length of preceding RR intervals in AF is strongly related to stroke volume [37]. LV ejection velocity is lower when pre-preceding RR intervals are longer, and differences in systolic performance are minimized when the preceding and pre-preceding RR interval lengths are similar [28]. Beat-to-beat variability in stroke volume increases as heart rate increases in AF patients [40], and the effect of preceding and pre-preceding RR intervals on stroke volume is most pronounced at higher heart rates [14].

With regard to the number of repeated measurements required, when preceding and pre-preceding RR interval lengths are equivalent (<60ms difference), measurement of PLSS in patients with persistent or permanent AF was similar using a single index-beat, as compared to averaging 15 cardiac cycles (r=0.97, p<0.001) [19]. In another study, index-beat assessment gave similar values to 10-second averages for myocardial strain and strain rate (r=0.94, p<0.001) [16]. The benefit of averaging a number of beats with similar preceding and pre-preceding RR

intervals and with cycle lengths of 500ms or greater was confirmed in two further studies [38, 39]. Using 3D volume datasets, a single-beat measurement in AF had lower variability than conventional 4-beat acquisition [29], although whether a single-beat analysis has the same association with clinical outcomes is currently unknown. For diastolic function, retest variability of E/e' was similar over 10 or 50 cardiac cycles in AF patients with preserved LVEF [20]. In another study of post-operative AF patients, the correlation of E/e' to PCWP was no different when sampling over 10 beats or in a single cycle with the longest RR interval (r= 0.47 and 0.44, respectively) [27].

These results suggest that choosing appropriate cardiac cycles with similar RR interval is more important than the absolute number of cycles measured (**Figure 2**). Of clinical importance, Nagueh *et al.* found less Doppler variability in patients at higher LV filling pressure [22], suggesting that measurement error might actually be reduced in those patients at the highest risk of adverse events.

Discussion

The main findings of this systematic review were that diastolic indices, in particular E/e’, were valid and reproducible in patients with AF, whereas data for systolic parameters were extremely limited. We also identified consensus amongst numerous studies that the optimal acquisition of echocardiography in AF patients occurred when preceding and pre-preceding cycle lengths are equivalent, rather than according to the number of repeated measurements taken. These findings have important clinical impact, dispelling preconceptions about the utility of diastolic variables, highlighting key areas in need of further prospective study, and improving the diagnostic value of echocardiography in patients with AF (**Figure 3**).

Echocardiography is a vital part of the assessment of AF patients, and is now recommend in all AF patients to guide management (class I, level of evidence C).[1] Numerous narrative reviews have been published concerning both systolic and diastolic function, however, this is the first systematic assessment of the validity and reproducibility of measurements. Echocardiography is an important component of initial management and is cost-effective for newly diagnosed patients with AF [41]. Knowledge about the type of heart failure in AF (preserved or reduced ejection fraction), has an important bearing on prognosis [42]. Identifying reduced LVEF also has consequences for the choice of rate- and rhythm-control therapy, for example the choice of beta-blockers or digoxin [43, 44], and the avoidance of non-dihydropyridine calcium channel blockers and class I anti-arrhythmic drugs. Echocardiography is also vital for the planning and follow-up of patients undergoing catheter, surgical and hybrid ablation for AF, as well as left atrial appendage closure.

As all of the studies were undertaken on patients in AF, the pooled data gives clinical guidance as to expected average values. The weighted-mean LVEF was 52.5%, and although a number of

studies either excluded or only enrolled those with heart failure, this was similar to the RealiseAF Global Registry (LVEF 54.3% in persistent and 53.3% in permanent AF) [4]. E/e' values were consistently higher than seen in 103 healthy volunteers (lateral E/e' 6.2±1.8 in age-range 60-69 years) [45] but similar to 100 sinus rhythm patients undergoing coronary angiography [46] and 951 sinus rhythm patients with isolated diastolic dysfunction and e'/a' <1. [47] However, even though average estimates are likely to be higher in AF patients (with associated comorbidities) than sinus rhythm, the cut-off value of E/e' >15 was still a good marker of adverse events and functional capacity in AF. Validation of E/e' against invasive filling pressure was reasonable in AF, and similar to correlation values published in sinus rhythm. For sinus rhythm, this includes lateral E/e′ r= 0.51 in 100 patients, lateral E/e' r= 0.86 in 100 patients, and septal E/e' r= 0.46 in 60 echocardiogram studies in 15 patients. [46, 48, 49] However, a recent systematic review of E/e' in sinus rhythm identified concerns over reliability of this parameter to estimate LV filling pressure.[50]

In all cases, there is the assumption that echocardiographic parameters are reliable in AF, despite the irregular ejection and rate. We have shown that stroke volume and LVEF do vary according to cycle length, particularly in respect to the RR intervals preceding measurement. In contrast to sinus rhythm, echocardiographers need to carefully appraise how and when to acquire measurements in order to accurately identify LV dysfunction in AF patients. Simultaneous assessment of both E and e' are now available in order to provide a single-beat analysis of E/e' (dual Doppler method). There are theoretical advantages to this process in reducing error, particularly in AF where successive beats are likely to vary. The dual Doppler method appears to offer better validation versus invasive PCWP (see **Table 4**), and in one study conferred a smaller amount of variability in E/e' between operators (7.1% versus 13.4% using conventional analysis over 10 cycles) [20]. However, it is unclear if this has any advantage over

properly acquired index-beat assessment, and availability in clinical practice is currently limited. Whereas a properly acquired index-beat assessment approach, based on our data, should achieve good levels of validity and reproducibility for diastolic indices, the data on systolic parameters is clearly inadequate. It is unclear which measure of systolic function is best for patients who are scanned whilst in AF, and this should be a priority for future research. Although global strain at a low cut-off was associated with outcomes in one of the studies reviewed [32], more recent data suggests that the association of strain with mortality is attenuated in patients with AF and heart failure with reduced LVEF (p value for interaction = 0.036) [51]. Further prospective studies, either in the context of controlled trials [52] or in routine clinical practice, are urgently needed to support the large volume of echocardiograms performed in patients with AF. As clinicians, we also need to know the minimum number of index beats required to maintain equivalence but reduce the time required for scanning, and for confirmation of reproducibility at different heart rates and grades of systolic and diastolic LV dysfunction.

Study limitations

There are numerous limitations to our review, most notably the risk of bias, particularly selection and blinding bias, as patients were often selected on the basis of echocardiogram quality. However, this is no different to studies in sinus rhythm. There are likely to be other studies assessing the reproducibility of echo parameters in AF, missed by our systematic search if reproducibility was not listed as a major outcome. We were unable to perform meta-analysis, not only because of the lack of published standard deviations for validation and reproducibility measures, but also the heterogeneity of populations assessed. Although most studies made reference to ‘chronic AF’, the duration and type of AF was often not disclosed. Most of the studies excluded valve disease (with differing definitions) and there was limited data above a heart rate of 100 beats/min. Finally, considering the importance of diagnosing heart failure in

patients with AF, and how common these conditions are in clinical practice, the relatively small number of studies identified in this systematic review is a surprising limitation, and one that requires further attention.

Conclusions

In selected patients with atrial fibrillation, diastolic echocardiographic parameters have been validated against invasive filling pressure, and E/e’ is an independent marker of functional impairment and adverse prognosis. Averaging single-beat assessments are reproducible and should be acquired in cycles with similar preceding length and controlled heart rate. However, data on the validity and reproducibility of systolic indices are extremely limited. Considering the importance of heart failure and assessment of systolic function in AF, further assessment of variability in routine clinical practice is urgently needed.

References

1. Kirchhof P, Benussi S, Kotecha D, Ahlsson A, Atar D, Casadei B, *et al.* 2016 ESC Guidelines for the management of atrial fibrillation developed in collaboration with EACTS. *Europace*. 2016;**18**:1609-1678

2. Kotecha D, Piccini JP. Atrial fibrillation in heart failure: what should we do? *Eur Heart J*. 2015;**36**:3250-3257

3. Kotecha D, Lam CS, Van Veldhuisen DJ, Van Gelder IC, Voors AA, Rienstra M. Heart Failure With Preserved Ejection Fraction and Atrial Fibrillation: Vicious Twins. *J Am Coll Cardiol*. 2016;**68**:2217-2228

4. Chiang CE, Naditch-Brule L, Murin J, Goethals M, Inoue H, O'Neill J, *et al.* Distribution and risk profile of paroxysmal, persistent, and permanent atrial fibrillation in routine clinical practice: insight from the real-life global survey evaluating patients with atrial fibrillation international registry. *Circ Arrhythm Electrophysiol*. 2012;**5**:632-639

5. Donal E, Lip GY, Galderisi M, Goette A, Shah D, Marwan M, *et al.* EACVI/EHRA Expert Consensus Document on the role of multi-modality imaging for the evaluation of patients with atrial fibrillation. *Eur Heart J Cardiovasc Imaging*. 2016;**17**:355-383

6. Lang RM, Badano LP, Mor-Avi V, Afilalo J, Armstrong A, Ernande L, *et al.* Recommendations for cardiac chamber quantification by echocardiography in adults: an update from the American Society of Echocardiography and the European Association of Cardiovascular Imaging. *Eur Heart J Cardiovasc Imaging*. 2015;**16**:233-270

7. Kim SY, Park JE, Lee YJ, Seo HJ, Sheen SS, Hahn S, *et al.* Testing a tool for assessing the risk of bias for nonrandomized studies showed moderate reliability and promising validity. *J Clin Epidemiol*. 2013;**66**:408-414

8. Belenkie I. Beat-to-beat variability of echocardiographic measurements of left ventricular end diastolic diameter and performance. *J Clin Ultrasound*. 1979;**7**:263-268

9. Benyounes N, Lang S, Soulat-Dufour L, Obadia M, Gout O, Chevalier G, *et al.* Can global longitudinal strain predict reduced left ventricular ejection fraction in daily echocardiographic practice? *Arch Cardiovasc Dis*. 2015;**108**:50-56

10. Chirillo F, Brunazzi MC, Barbiero M, Giavarina D, Pasqualini M, Franceschini-Grisolia E, *et al.* Estimating mean pulmonary wedge pressure in patients with chronic atrial fibrillation from transthoracic Doppler indexes of mitral and pulmonary venous flow velocity. *J Am Coll Cardiol*. 1997;**30**:19-26

11. Diwan A, McCulloch M, Lawrie GM, Reardon MJ, Nagueh SF. Doppler Estimation of Left Ventricular Filling Pressures in Patients With Mitral Valve Disease. *Circulation*. 2005;**111**:3281-3289

12. Dubrey SW, Falk RH. Optimal number of beats for the Doppler measurement of cardiac output in atrial fibrillation. *J Am Soc Echocardiogr*. 1997;**10**:67-71

13. Galderisi M, Benjamin EJ, Evans JC, D'Agostino RB, Fuller DL, Lehman B, *et al.* Intra- and interobserver reproducibility of Doppler-assessed indexes of left ventricular diastolic function in a population-based study (the Framingham Heart Study). *Am J Cardiol*. 1992;**70**:1341-1346

14. Kerr AJ, Simmonds MB, Stewart RA. Influence of heart rate on stroke volume variability in atrial fibrillation in patients with normal and impaired left ventricular function. *Am J Cardiol*. 1998;**82**:1496-1500

15. Kusunose K, Yamada H, Nishio S, Tomita N, Niki T, Yamaguchi K, *et al.* Clinical utility of single-beat E/e' obtained by simultaneous recording of flow and tissue Doppler velocities in atrial fibrillation with preserved systolic function. *JACC Cardiovasc Imaging*. 2009;**2**:1147-1156

16. Kusunose K, Yamada H, Nishio S, Tomita N, Hotchi J, Bando M, *et al.* Index-beat assessment of left ventricular systolic and diastolic function during atrial fibrillation using myocardial strain and strain rate. *J Am Soc Echocardiogr*. 2012;**25**:953-959

17. Lee SH, Jung JH, Choi SH, Lee N, Oh DJ, Ryu KH, *et al.* Exercise intolerance in patients with atrial fibrillation: clinical and echocardiographic determinants of exercise capacity. *J Am Soc Echocardiogr*. 2005;**18**:1349-1354

18. Lee SH, Choi S, Chung WJ, Byun YS, Ryu SK, Pyun WB, *et al.* Tissue Doppler index, E/E', and ischemic stroke in patients with atrial fibrillation and preserved left ventricular ejection fraction. *J Neurol Sci*. 2008;**271**:148-152

19. Lee CS, Lin TH, Hsu PC, Chu CY, Lee WH, Su HM, *et al.* Measuring left ventricular peak longitudinal systolic strain from a single beat in atrial fibrillation: validation of the index beat method. *J Am Soc Echocardiogr*. 2012;**25**:945-952

20. Li C, Zhang J, Zhou C, Huang L, Tang H, Rao L. Will simultaneous measurement of E/e' index facilitate the non-invasive assessment of left ventricular filling pressure in patients with non-valvular atrial fibrillation? *Eur J Echocardiogr*. 2010;**11**:296-301

21. Matsukida K, Kisanuki A, Toyonaga K, Murayama T, Nakashima H, Kumanohoso T, *et al.* Comparison of transthoracic Doppler echocardiography and natriuretic peptides in predicting mean pulmonary capillary wedge pressure in patients with chronic atrial fibrillation. *J Am Soc Echocardiogr*. 2001;**14**:1080-1087

22. Nagueh SF, Kopelen HA, Quinones MA. Assessment of left ventricular filling pressures by Doppler in the presence of atrial fibrillation. *Circulation*. 1996;**94**:2138-2145

23. Okura H, Takada Y, Kubo T, Iwata K, Mizoguchi S, Taguchi H, *et al.* Tissue Doppler-derived index of left ventricular filling pressure, E/E', predicts survival of patients with non-valvular atrial fibrillation. *Heart*. 2006;**92**:1248-1252

24. Oyama R, Murata K, Tanaka N, Takaki A, Ueda K, Liu J, *et al.* Is the ratio of transmitral peak E-wave velocity to color flow propagation velocity useful for evaluating the severity of heart failure in atrial fibrillation? *Circ J*. 2004;**68**:1132-1138

25. Peltier M, Leborgne L, Zoubidi M, Slama M, Tribouilloy CM. Prognostic value of short-deceleration time of mitral inflow E velocity: implications in patients with atrial

fibrillation and left-ventricular systolic dysfunction. *Arch Cardiovasc Dis*. 2008;**101**:317-325

26. Punjani S, Wu WC, Cohen S, Sharma SC, Choudhary G. Echocardiographic indices of diastolic function relate to functional capacity and quality of life in ambulatory men with atrial fibrillation. *J Am Soc Echocardiogr*. 2011;**24**:533-540 e533

27. Senechal M, O'Connor K, Deblois J, Magne J, Dumesnil JG, Pibarot P*, et al.* A simple Doppler echocardiography method to evaluate pulmonary capillary wedge pressure in patients with atrial fibrillation. *Echocardiography*. 2008;**25**:57-63

28. Schneider F, Martin DT, Schick EC, Gaasch WH. Interval-dependent changes in left ventricular contractile state in lone atrial fibrillation and in atrial fibrillation associated with coronary artery disease. *Am J Cardiol*. 1997;**80**:586-590

29. Shahgaldi K, Manouras A, Abrahamsson A, Gudmundsson P, Brodin LA, Winter R. Three-dimensional echocardiography using single-heartbeat modality decreases variability in measuring left ventricular volumes and function in comparison to four-beat technique in atrial fibrillation. *Cardiovasc Ultrasound*. 2010;**8**:45

30. Sohn DW, Song JM, Zo JH, Chai IH, Kim HS, Chun HG*, et al.* Mitral annulus velocity in the evaluation of left ventricular diastolic function in atrial fibrillation. *J Am Soc Echocardiogr*. 1999;**12**:927-931

31. Su HM, Lin TH, Hsu PC, Chu CY, Lee WH, Lee CS*, et al.* Myocardial performance index derived from preejection period: a novel and feasible parameter in evaluation of cardiac performance in patients with permanent atrial fibrillation. *Echocardiography*. 2011;**28**:1081-1087

32. Su HM, Lin TH, Hsu PC, Lee WH, Chu CY, Lee CS*, et al.* Global left ventricular longitudinal systolic strain as a major predictor of cardiovascular events in patients with atrial fibrillation. *Heart*. 2013;**99**:1588-1596

33. Thavendiranathan P, Liu S, Verhaert D, Calleja A, Nitinunu A, Van Houten T*, et al.* Feasibility, accuracy, and reproducibility of real-time full-volume 3D transthoracic echocardiography to measure LV volumes and systolic function: a fully automated endocardial contouring algorithm in sinus rhythm and atrial fibrillation. *JACC Cardiovasc Imaging*. 2012;**5**:239-251

34. Temporelli PL, Scapellato F, Corra U, Eleuteri E, Imparato A, Giannuzzi P. Estimation of pulmonary wedge pressure by transmitral Doppler in patients with chronic heart failure and atrial fibrillation. *Am J Cardiol*. 1999;**83**:724-727

35. Traversi E, Cobelli F, Pozzoli M. Doppler echocardiography reliably predicts pulmonary artery wedge pressure in patients with chronic heart failure even when atrial fibrillation is present. *Eur J Heart Fail*. 2001;**3**:173-181

36. Wada Y, Murata K, Tanaka T, Nose Y, Kihara C, Uchida K*, et al.* Simultaneous Doppler tracing of transmitral inflow and mitral annular velocity as an estimate of elevated left ventricular filling pressure in patients with atrial fibrillation. *Circ J*. 2012;**76**:675-681

37. Wang CL, Lin KH, Luqman N, Chu PH, Hsu LA, Kuo CT. Simultaneous biplane single-

beat assessment of left ventricular systolic function in patients with atrial fibrillation. *Am J Cardiol*. 2004;**94**:942-944

38. Wang CL, Lin KH, Luqman N, Ho WJ, Hsu LA, Chu PH, *et al.* Refinement on single-beat determination of left ventricular systolic function in patients with atrial fibrillation. *J Am Soc Echocardiogr*. 2005;**18**:913-918

39. Wang CL, Ho WJ, Luqman N, Hsu LA, Kuo CT. Biplane assessment of left ventricular function during atrial fibrillation at beats with equal subsequent cycles. *Int J Cardiol*. 2006;**113**:54-60

40. Ko HS, Kim CJ, Ryu WS. New parameters for left ventricular function in atrial fibrillation: based on the relationship between RR interval and performance. *J Korean Med Sci*. 2005;**20**:20-25

41. Simpson E, Stevenson M, Scope A, Poku E, Minton J, Evans P. Echocardiography in newly diagnosed atrial fibrillation patients: a systematic review and economic evaluation. *Health Technol Assess*. 2013;**17**:1-263, v-vi

42. Kotecha D, Chudasama R, Lane DA, Kirchhof P, Lip GY. Atrial fibrillation and heart failure due to reduced versus preserved ejection fraction: A systematic review and meta-analysis of death and adverse outcomes. *Int J Cardiol*. 2016;**203**:660-666

43. Kotecha D, Holmes J, Krum H, Altman DG, Manzano L, Cleland JG, *et al.* Efficacy of beta blockers in patients with heart failure plus atrial fibrillation: an individual-patient data meta-analysis. *Lancet*. 2014;**384**:2235-2243

44. Ziff OJ, Lane DA, Samra M, Griffith M, Kirchhof P, Lip GY, *et al.* Safety and efficacy of digoxin: systematic review and meta-analysis of observational and controlled trial data. *BMJ*. 2015;**351**:h4451

45. Tighe DA, Vinch CS, Hill JC, Meyer TE, Goldberg RJ, Aurigemma GP. Influence of age on assessment of diastolic function by Doppler tissue imaging. *Am J Cardiol*. 2003;**91**:254-257

46. Ommen SR, Nishimura RA, Appleton CP, Miller FA, Oh JK, Redfield MM, *et al.* Clinical utility of Doppler echocardiography and tissue Doppler imaging in the estimation of left ventricular filling pressures: A comparative simultaneous Doppler-catheterization study. *Circulation*. 2000;**102**:1788-1794

47. Kim SA, Park WJ, Kim HJ, Jo SH, Kim HS, Han SJ, *et al.* Determinants of preserved diastolic function at the lateral annulus in patients with isolated diastolic dysfunction. *Eur Heart J Cardiovasc Imaging*. 2013;**14**:443-448

48. Nagueh SF, Mikati I, Kopelen HA, Middleton KJ, Quinones MA, Zoghbi WA. Doppler estimation of left ventricular filling pressure in sinus tachycardia. A new application of tissue doppler imaging. *Circulation*. 1998;**98**:1644-1650

49. Ritzema JL, Richards AM, Crozier IG, Frampton CF, Melton IC, Doughty RN, *et al.* Serial Doppler echocardiography and tissue Doppler imaging in the detection of elevated directly measured left atrial pressure in ambulant subjects with chronic heart failure. *JACC Cardiovasc Imaging*. 2011;**4**:927-934

50. Sharifov OF, Schiros CG, Aban I, Denney TS, Gupta H. Diagnostic Accuracy of Tissue Doppler Index E/e' for Evaluating Left Ventricular Filling Pressure and Diastolic Dysfunction/Heart Failure With Preserved Ejection Fraction: A Systematic Review and Meta-Analysis. *J Am Heart Assoc*. 2016;**5**

51. Sengelov M, Jorgensen PG, Jensen JS, Bruun NE, Olsen FJ, Fritz-Hansen T*, et al.* Global Longitudinal Strain Is a Superior Predictor of All-Cause Mortality in Heart Failure With Reduced Ejection Fraction. *JACC Cardiovasc Imaging*. 2015;**8**:1351-1359

52. Institute of Cardiovascular Sciences; University of Birmingham, Chief Investigator Kotecha D. Rate Control Therapy Evaluation in Permanent Atrial Fibrillation (RATE-AF). *National Library of Medicine (US)*. 2015; **NLM Identifier: NCT02391337 [Accessed 20-Nov-2015]**: Available from: https://clinicaltrials.gov/ct2/show/NCT02391337

Conflicts

All authors have completed the ICMJE conflict of interest statement and report no conflicts. DK discloses non-financial support from Daiitchi Sankyo, research grants from Menarini and lecture fees from AtriCure, all outside the submitted work; and Lead for the Beta-blockers in Heart Failure Collaborative Group (BB-meta-HF) and the RAte control Therapy Evaluation in Atrial Fibrillation (RATE-AF) trial. MM, ES and RS have no disclosures to report. BAP discloses research support and honoraria from GE Healthcare and Hitachi-Aloka, all outside the submitted work.

Funding

DK is supported by a National Institute of Health Research (NIHR) Career Development Fellowship (CDF-2015-08-074). The opinions expressed in this paper are those of the authors and do not represent the NIHR or the UK Department of Health.

Statement

The manuscript has neither been published (except in the form of abstract or thesis) nor is currently under consideration for publication by any other journal. All named authors have seen and approved the final version of the manuscript.

Table 1: Summary of included studies

Study	Number with AF	Population	Relevant topic(s)	Aims and methods	Main findings related to AF
**Belenkie, 1979** [8]	11	Patients with sinus rhythm and AF, excluding technically inadequate echocardiograms.	Acquisition.	Association of end diastolic dimension and cycle length with M-mode parameters of LV systolic function.	Preload and cycle length correlated with LVEF. Patients with AF had higher correlation of RR interval with LVEF than patients with sinus rhythm.
**Benyounes, 2015** [9]	17	Consecutive patients including those with AF, but no important variability in heart rhythm.	Systolic validity.	Internal validation of strain measurement against LVEF.	High correlation of strain and LVEF in AF patients, and probably similar to sinus rhythm.
**Chirillo, 1997** [10]	35	AF for >3 months without mitral stenosis, undergoing catheterization on intensive care or electively.	Diastolic validity.	Correlation of invasive PCWP with mitral and PV flow velocities and derivation of non-invasive algorithm.	Diastolic PV flow better than mitral indices for estimating PCWP in AF.
**Diwan, 2005** [11]	13	Consecutive patients with mitral valve disease undergoing catheterization.	Diastolic validity.	Correlation of invasive PCWP with Doppler indices of diastolic function.	The ratio of IVRT to the time period between E and e' highly correlated with PCWP in AF, similar to sinus rhythm.
**Dubrey, 1997** [12]	21	Selected AF patients with irregularity of rate on electrocardiogram.	Systolic reproducibility and acquisition.	Variability in LV outflow tract Doppler in AF compared to sinus rhythm.	13 beats required in AF to achieve variability <2%, compared to 4 beats in sinus rhythm.
**Galderisi, 1992** [13]	12	Patients randomly selected from the Framingham cohort with heart rate <100 beats/min and technically adequate Doppler.	Diastolic reproducibility.	Reproducibility of Doppler indices of diastolic function in sinus rhythm and AF.	Variability similar in AF and sinus rhythm. Reproducibility highest for peak velocity and area under the curve rather than slope measures.
**Kerr, 1998** [14]	38	Consecutive non-valvular AF patients with good quality echocardiography.	Acquisition.	Impact of heart rate cycle length variability on LV outflow tract Doppler.	Variability in stroke volume increased at higher heart rates in AF.
**Kusunose, 2009** [15]	56	Non-valvular AF patients with preserved systolic function (n=21 with simultaneous catheterization).	Diastolic validity and reproducibility.	Validation of single-beat E/e' recorded by synchronous dual Doppler.	Single-beat lateral E/e' a reliable indicator of elevated PCWP and plasma BNP in AF patients.
**Kusunose, 2012** [16]	25	Prospective assessment of non-valvular AF patients referred for catheterization.	Systolic reproducibility and acquisition.	Validation of an index-beat assessment versus 10-second average for myocardial strain and strain rate.	A single index-beat (with similar preceding and pre-preceding RR intervals) was accurate compared to averaged mean values.
**Lee, 2005** [17]	73	Non-valvular chronic AF with heart rate ≤100 beats/min and clinically stable.	Systolic and diastolic validity.	Correlation of clinical and echocardiographic parameters with maximum symptom-limited treadmill.	E/e' significantly related to exercise capacity in AF, unlike other echocardiographic parameters.

Study	Number with AF	Population	Relevant topic(s)	Aims and methods	Main findings related to AF
**Lee, 2008** [18]	330	Multicentre consecutive patients with persistent AF, LVEF >40% and heart rate ≤100 beats/min.	Systolic and diastolic validity.	Identification of echocardiographic risk factors for retrospective ischemic stroke.	E/e' significantly associated with prior stroke in AF patients with LVEF >40%.
**Lee, 2012** [19]	98	Prospective study of persistent or permanent AF patients with heart rate ≤105 beats/min.	Systolic reproducibility and acquisition.	Validation of index-beat measurement of LV peak longitudinal systolic strain.	A single index-beat was accurate compared to averaging multiple cardiac cycles.
**Li, 2010** [20]	49	Non-valvular AF patients with preserved ejection fraction undergoing catheterization.	Diastolic validity and reproducibility.	Correlation of single-beat E/e' with invasive PCWP.	Stronger association between E/e' and PCWP using a single-beat, dual Doppler method.
**Matsukida, 2001** [21]	32	Chronic AF patients undergoing catheterization.	Diastolic validity and reproducibility.	Correlation of diastolic indices with invasive PCWP.	PV flow and deceleration time independently associated with PCWP.
**Nageuh, 1996** [22]	60	Non-valvular AF patients (majority intensive care or surgical patients).	Diastolic validity and reproducibility.	Correlation of diastolic indices with invasive PCWP in training and test groups.	Diastolic indices (e.g. IVRT) highly correlated with PCWP, particularly in AF patients with LVEF <45%.
**Okura, 2006** [23]	230	Retrospective analysis of consecutive non-valvular AF patients.	Diastolic validity and reproducibility.	Assessment of E/e' at a cut-point of 15 as a predictor of mortality over a follow-up period of 245 (± 200) days.	AF patients with E/e' >15 have higher mortality, independent of clinical factors.
**Oyama, 2004** [24]	68	Non-valvular chronic AF patients.	Diastolic validity and reproducibility.	Correlation of E/Vp using single-beat dual Doppler with plasma BNP concentration and invasive PCWP.	E/Vp associated with both BNP and PCWP.
**Peltier, 2008** [25]	40	Prospective assessment of patients with non-valvular AF >1 month and LVEF <40%, hospitalized for heart failure.	Diastolic validity and reproducibility.	Correlation of E/e' with functional capacity and quality of life.	Deceleration time <150ms was independently associated with mortality in both AF and sinus rhythm.
**Punjani, 2011** [26]	48	Retrospective analysis of persistent or permanent AF with LVEF ≥50% and heart rate ≤100 beats/min.	Diastolic validity and reproducibility.	Determine relationship between diastolic parameters and functional capacity/quality of life, when measured on two different occasions 1 week apart.	E/e' independently associated with walk distance and quality of life in patients with AF and preserved LVEF.
**Schneider, 1997** [28]	18	Chronic AF patients during routine echocardiography.	Acquisition.	Test hypothesis that LV systolic function is affected by pre-preceding cycle length.	Pre-preceding RR interval has an important effect on LV peak ejection velocity.
**Senechal, 2008** [27]	24	Consecutive early post-operative patients with predominantly paroxysmal AF and no mitral prosthesis.	Diastolic validity, diastolic reproducibility and acquisition.	E/e' for estimating invasive PCWP with comparison of 10-beat average and one cycle with the longest R-R interval.	E/e' with a single cardiac cycle had similar correlation with PCWP as averaged measures.

Study	Number with AF	Population	Relevant topic(s)	Aims and methods	Main findings related to AF
**Shahgaldi, 2010** [29]	23	Consecutive patients referred for echocardiography.	Systolic reproducibility.	Comparison of 1-beat and 4-beat 3D volumes and LVEF in patients with sinus rhythm and AF.	Lower variability in 3D full volume acquisition in AF patients using a 1-beat rather than 4-beat acquisition.
**Sohn, 1999** [30]	27	Non-valvular AF patients undergoing catheterization.	Diastolic validity.	Correlation of E/e' with invasive PCWP.	E/e' highly correlated with PCWP.
**Su, 2011** [31]	54	Consecutive patients with permanent AF and adequate echocardiographic images.	Systolic reproducibility.	Validation of pre-ejection period myocardial performance index with other indices of systolic and diastolic function in AF.	Pre-ejection period myocardial performance index is an indicator of global LV function in permanent AF.
**Su, 2013** [32]	196	Prospective assessment of consecutive patients with persistent AF and adequate images.	Systolic validity and reproducibility.	Ability of global longitudinal strain to predict cardiovascular events over follow-up of 21 (±10) months.	Global longitudinal strain improved prediction of adverse events beyond LVEF and tissue Doppler assessment.
**Temporelli, 1999** [34]	35	Patients with heart failure, LVEF <35%, AF >3 months and acceptable images.	Diastolic validity and reproducibility.	Correlation of diastolic indices with invasive PCWP.	Deceleration time was independently associated with PCWP in AF patients with severe LV dysfunction.
**Thavendiranthan, 2012** [33]	24	Subgroup of patients with AF referred for an echocardiogram (main study outcomes investigated patients with sinus rhythm).	Systolic validation.	Assessment of an automated edge contouring algorithm using real-time 3D acquisition, compared to conventional biplane Simpsons.	Automated 3D LVEF in AF patients correlated well with conventional LVEF analysis.
**Traversi, 2001** [35]	51	Patients with heart failure, LVEF <35%, AF >3 months and heart rate <90 beats/min, as part of a pre-transplant evaluation.	Diastolic validity and reproducibility.	Correlation of diastolic indices with invasive PCWP.	Mitral and PV flow indices correlate with PCWP in AF patients assessed for heart transplantation.
**Wada, 2012** [36]	45	Non-valvular chronic AF patients with normal right ventricular function.	Diastolic validity and reproducibility.	Correlation of single-beat dual Doppler with invasive PCWP.	The time and ratio between E and e' correlated with PCWP, similar to BNP.
**Wang, 2004** [37]	40	Consecutive patients with AF and adequate acoustic windows.	Acquisition.	Evaluation of LVEF and stroke volume according to preceding cycle lengths.	Positive relationship between preceding cycle length and stroke volume.
**Wang, 2005** [38]	100	Consecutive AF patients referred for echocardiogram with adequate acoustic windows.	Acquisition.	Evaluation of aortic time-velocity integral according to preceding cycle length and varying beat repeats.	Assessment improved with cycle lengths >500ms and 2 or 3 beats with similar RR interval.
**Wang, 2006** [39]	75	Patients with AF referred for echocardiography with adequate acoustic windows.	Systolic reproducibility and acquisition	Improvement of systolic function evaluation according to cycle lengths and number of repeated beats.	LVEF and stroke volume can be reliably obtained in AF by averaging two beats with similar preceding and pre-preceding RR intervals and cycle length >500ms.

3D = Three-dimensional; AF = atrial fibrillation; BNP = B-type natriuretic peptide; IVRT = isovolumic relaxation time; LV = left ventricular; LVEF = left ventricular ejection fraction; PCWP = pulmonary capillary wedge pressure; PV = pulmonary vein.

Table 2: Pooled characteristics

Characteristic	Range of reported means	Weighted average (standard deviation of means)	Number of studies / number of patients
Age	57 - 76 years	66.9 (4.5) years	31 / 1916
Women	0 – 52 %	33 (11) %	27 / 1835
Hypertension	17 - 85 %	53 (18) %	11 / 1235
Heart failure	0 - 100 %	48 (35) %	14 / 1473
LVEF	22 - 65 %	52.5 (9.7) %	25 / 1646
E/e’:			
Average	9 – 23	11.7 (2.7)	5 / 437
Septal	11 – 23	13.4 (4.7)	2 / 560
Lateral	8 – 14	10.3 (2.1)	5 / 196
Heart rate	63 - 107 beats/min	79.9 (6.3) beats/min	20 / 1223

Pooled baseline characteristics, weighted according to sample size. E/e’ = ratio of mitral peak E velocity and tissue Doppler early diastolic filling e’; LVEF = left ventricular ejection fraction.

Table 3: Reproducibility of systolic echocardiographic measures in AF

Parameter / Study	N	REPRODUCIBILITY<br>Intra-observer and inter-observer variability
**Simpson's LVEF:**<br>Wang, 2006 [39]	10	Single-beat intra 2.8%
**3-dimensional LVEF:**<br>Shahgaldi, 2010 [29]	23	4-beat intra 8.3%, inter 17.9%<br>Single beat intra 4.8%, inter 5.6%
**Peak longitudinal systolic strain:**<br>Lee, 2012 [19]	15	15-cycle average intra 2.4%, inter 2.7%<br>Single index beat intra 3.5%, inter 4.0%
**Global longitudinal strain*:**<br>Su, 2013 [32]	30	Intra 5.3%, inter 6.2%
**Myocardial performance index†:**<br>Su, 2011 [31]	54	Intra 5.2%, inter 7.3%

LVEF = left ventricular ejection fraction; * Using single index beat; † A marker of combined systolic and diastolic function calculated as the sum of pre-ejection time and isovolumic relaxation time as a ratio of ejection time.

Table 4: Validity and reproducibility of diastolic echocardiographic measures in AF

		**DIASTOLIC VALIDATION**	**DIASTOLIC REPRODUCIBILITY**	
**Parameter / Study**	**N**	**Correlation with invasive pulmonary capillary wedge pressure (r)**	**Intra-observer and inter-observer mean differences (MD) ± standard deviation, coefficient of variation (CV), retest correlation (RC) or retest variability (RV)**	**Mean LVEF (SD) %**
**Isovolumic relaxation time (IVRT):**				
Nagueh, 1996 [22]	30	-0.76[‡]	Intra MD 1.4±8.4ms, inter MD 4.5±9.0ms[‖]	45 (16)
Temporelli, 1999 [34]	35	-0.95[‡]	CV 1.9-2.4%[¶]	22 (5)
Traversi, 2001 [35]	51	-0.70[‡]	Intra MD 0.15±0.15, inter MD 0.25±1.64mmHg[††]	25 (7)
Diwan, 2005 [11]	13	-0.92[†,§]		54 (11)
Punjani, 2011 [26]	48		Intra RC 0.54	
**Mitral E wave deceleration time:**				
Galderisi, 1992 [13]	12		Intra RC 0.85-0.93, inter RC 0.76	
Nagueh, 1996 [22]	30	-0.42*	Intra MD 1.0±4.0ms; inter MD 5.4±7.8ms[‖]	45 (16)
Chirillo, 1997 [10]	35	-0.50[†]	CV "not statistically significant"	41 (13)
Sohn, 1999 [30]	27	no correlation		53 (11)
Temporelli, 1999 [34]	35	-0.70[†]	CV 1.9-2.4%[¶]	22 (5)
Matsukida, 2001 [21]	32	-0.65[‡]	Intra RV 5.1%, inter RV 5.6%[¶]	[‡‡]
Traversi, 2001 [35]	51	-0.60[‡]		25 (7)
Peltier, 2008 [25]	30		Intra RC 0.88, inter RC 0.84.	31 (8)
Senechal, 2008 [27]	24	no correlation	Intra RV 1.2-3.6%, inter RV 2.3-4.8%[¶], **	46 (15)
Punjani, 2011 [26]	48		Intra RC 0.75	
**Ratio of mitral peak E velocity and tissue Doppler e' (E/e'):**				
Sohn, 1999 [30]	27	Septal 0.79[‡]		53 (11)
Okura, 2006 [23]	230		Septal intra RV 5.0%, inter RV 11.4%	56 (12)
Senechal, 2008 [27]	24	Lateral 0.47*, septal 0.46*	Intra RV 1.2-3.6%, inter RV 2.3-4.8%[¶], **	46 (15)
Kusunose, 2009 [15]	21	Lateral 0.57[†], single-beat lateral 0.74[‡]	Single-beat lateral intra RV 4.9%, inter RV 6.6%[#]	60 (6)
Li, 2010 [20]	49	Lateral 0.49[‡], single-beat lateral 0.77[‡]	Single-beat lateral intra RV 6.7%, inter RV 7.9%	59 (8)
Punjani, 2011 [26]	48		Lateral intra RC 0.84, septal intra RC 0.86	
Wada, 2012 [36]	45	Average single-beat 0.57[‡]	Single-beat average intra RV 4.3%, inter RV 11.1%	52 (16)
**Ratio of mitral peak E velocity and velocity of diastolic flow propagation (E/Vp):**				
Nagueh, 1996 [22]	30	0.65[‡]	Intra MD 0.2±0.4ms, inter MD 0.13±0.40ms[‖]	45 (16)
Oyama, 2004 [24]	25	0.63[†]	Intra RV 5.1%, inter 5.3%	55 (15)
Punjani, 2011 [26]	48		Intra RC 0.79	
**Pulmonary venous flow diastolic wave deceleration time (PVd-DT):**				
Chirillo, 1997 [10]	35	-0.91[‡]	CV "not statistically significant"	41 (13)
Matsukida, 2001 [21]	32	-0.80[‡]	Intra RV 5.1%, inter RV 5.6%[¶]	[‡‡]

LVEF = left ventricular ejection fraction. * p≤0.05; [†] p≤0.01; [‡] p<0.001; [§] IVRT as a ratio to the difference between onset time of mitral E and annulus e' velocities; [‖] N=60 for reproducibility data; [¶] Combined reproducibility assessment for all Doppler variables; [#] N=10 for reproducibility data; ** N=6 for reproducibility data; [††] N=40 for reproducibility data; based on a composite of IVRT, deceleration rate and systolic fraction; [‡‡] Fractional shortening 29% (SD 4%). Retest variability typically expressed as the mean percentage error.

Supplementary Table A: Risk of Bias assessment

Study	Selection of participants	Confounding variables	Measurement of exposure	Blinding of outcome	Incomplete outcome data	Selective reporting
Belenkie, 1979 [8]	High	High	High	High	Low	Low
Benyounes, 2015 [9]	High	High	Low	High	High	Unclear
Chirillo, 1997 [10]	Low	Low	Unclear	High	Low	Low
Diwan, 2005 [11]	Unclear	Unclear	Low	High	High	Unclear
Dubrey, 1997 [12]	Low	Low	High	High	Unclear	Unclear
Galderisi, 1992 [13]	Low	Low	Low	High	Low	Low
Kerr, 1998 [14]	Low	Low	Low	Low	Low	Unclear
Kusunose, 2009 [15]	Low	Low	Low	High	Low	Low
Kusunose, 2012 [16]	High	Low	Low	High	Low	Low
Lee, 2005 [17]	High	Low	High	High	High	Unclear
Lee, 2008 [18]	Low	Unclear	High	High	Unclear	Unclear
Lee, 2012 [19]	High	Low	High	High	Unclear	Low
Li, 2010 [20]	High	Low	Low	High	Unclear	Unclear
Matsukida, 2001 [21]	Low	Low	Low	Unclear	Unclear	Unclear
Nageuh, 1996 [22]	Unclear	High	Unclear	High	Low	Low
Okura, 2006 [23]	High	Low	Low	High	Unclear	Unclear
Oyama, 2004 [24]	Unclear	High	High	High	High	Unclear
Peltier, 2008 [25]	High	Low	Unclear	Low	Low	Unclear
Punjani, 2011 [26]	High	High	High	Low	Low	Low
Schneider, 1997 [28]	High	High	High	High	Low	High
Senechal, 2008 [27]	High	Unclear	High	High	Low	Unclear
Shahgaldi, 2010 [29]	Low	Low	Low	Low	Unclear	Low
Sohn, 1999 [30]	High	High	High	High	Unclear	Unclear
Su, 2011 [31]	High	Low	High	High	Low	Unclear
Su, 2013 [32]	Low	Low	Low	Low	Low	Low
Temporelli, 1999 [34]	High	Low	High	High	High	High
Thavendiranthan, 2012 [33]	High	Low	Low	Low	Unclear	Low
Traversi, 2001 [35]	High	High	Low	High	Unclear	Unclear
Wada, 2012 [36]	High	Low	High	High	Unclear	Unclear
Wang, 2004 [37]	High	Low	High	High	Unclear	Unclear
Wang, 2005 [38]	High	Low	High	High	Unclear	Unclear
Wang, 2006 [39]	High	Low	High	High	Unclear	Unclear

Figure legends

Figure 1: Systematic review flowchart

AF = atrial fibrillation.

Figure 2: Example of optimal acquisition (index beat method)

In order to achieve the most valid and reproducible measurement in atrial fibrillation, parameters should be acquired where the two preceding cardiac cycles have similar RR-intervals and preferably where the equivalent heart rate is <100 beats/minute (panel A). This method can also be applied to assessment of function; averaging individual index beats is preferable to averaging across sequential cardiac cycles (panel B).

Figure 3: Summary of findings for echocardiography in AF

AF = atrial fibrillation; E/e’ = ratio of mitral peak E velocity and tissue Doppler early diastolic filling e’; E/Vp = ratio of mitral peak E velocity and the velocity of diastolic flow propagation; IVRT = isovolumic relaxation time; PCWP = pulmonary capillary wedge pressure; PVd-DT = pulmonary venous diastolic flow deceleration time.

Figure 1

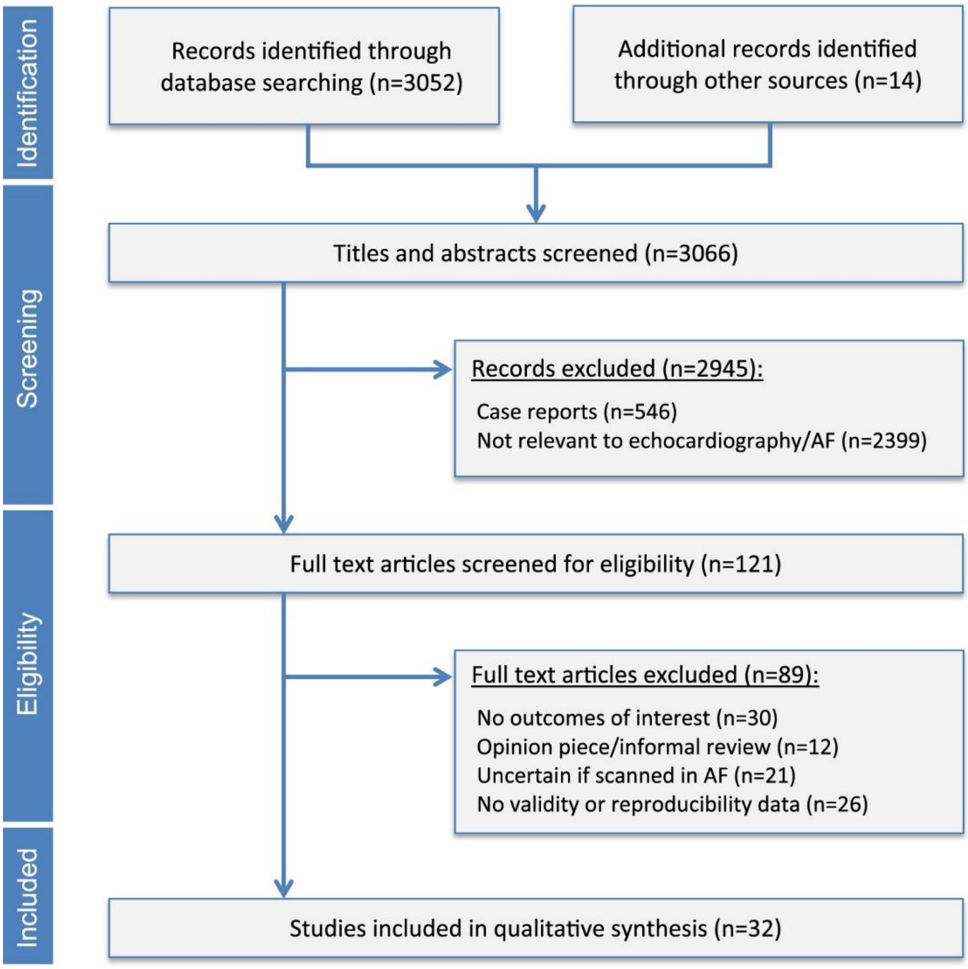

Identification
Screening
Eligibility
Included
Records identified through database searching (n=3052)
Additional records identified through other sources (n=14)
Titles and abstracts screened (n=3066)
Records excluded (n=2945):
Case reports (n=546)
Not relevant to echocardiography/AF (n=2399)
Full text articles screened for eligibility (n=121)
Full text articles excluded (n=89):
No outcomes of interest (n=30)
Opinion piece/informal review (n=12)
Uncertain if scanned in AF (n=21)
No validity or reproducibility data (n=26)
Studies included in qualitative synthesis (n=32)

Figure 2

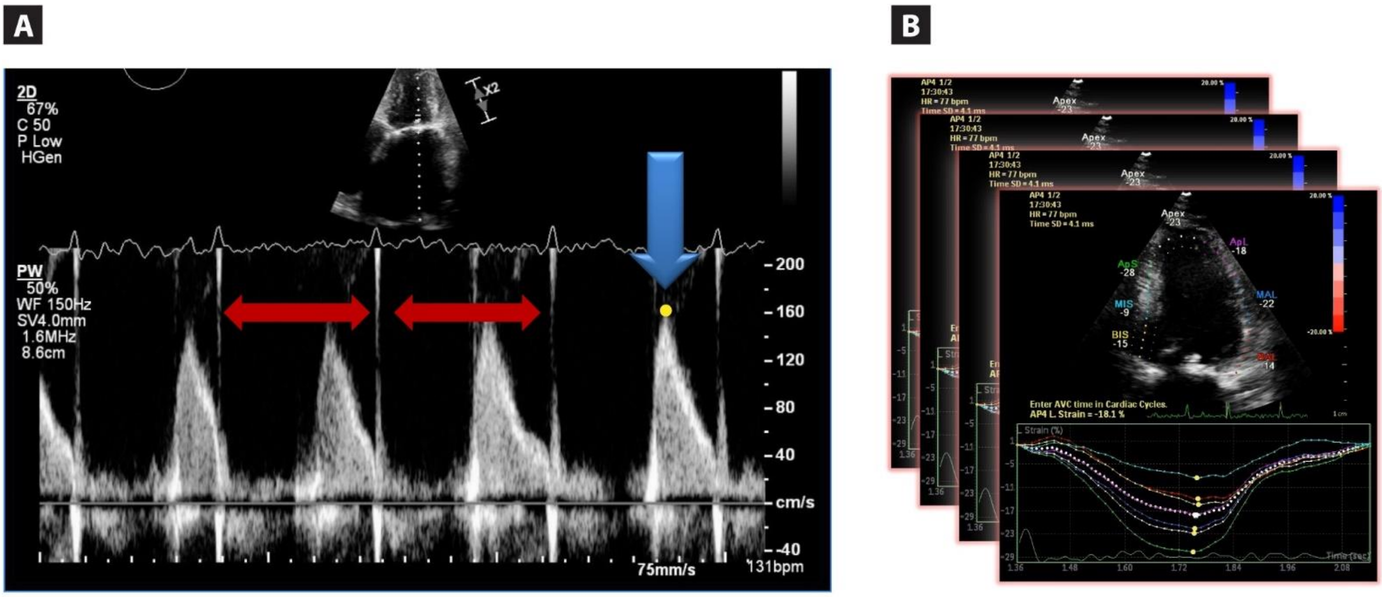
A
B
2D
67%
C 50
P Low
HGen
PW
50%
WF 150Hz
SV4.0mm
1.6MHz
8.6cm
-200
-160
-120
-80
-40
cm/s
-40
75mm/s
131bpm
AP4 1/2
17:30:43
HR = 77 bpm
Time SD = 4.1 ms
Apex
-23
ApL
-18
ApS
-28
MAL
-22
MIS
-9
BIS
-15
Enter AVC time in Cardiac Cycles.
AP4 L. Strain = -18.1 %
Strain (%)
Time (sec)

Figure 3

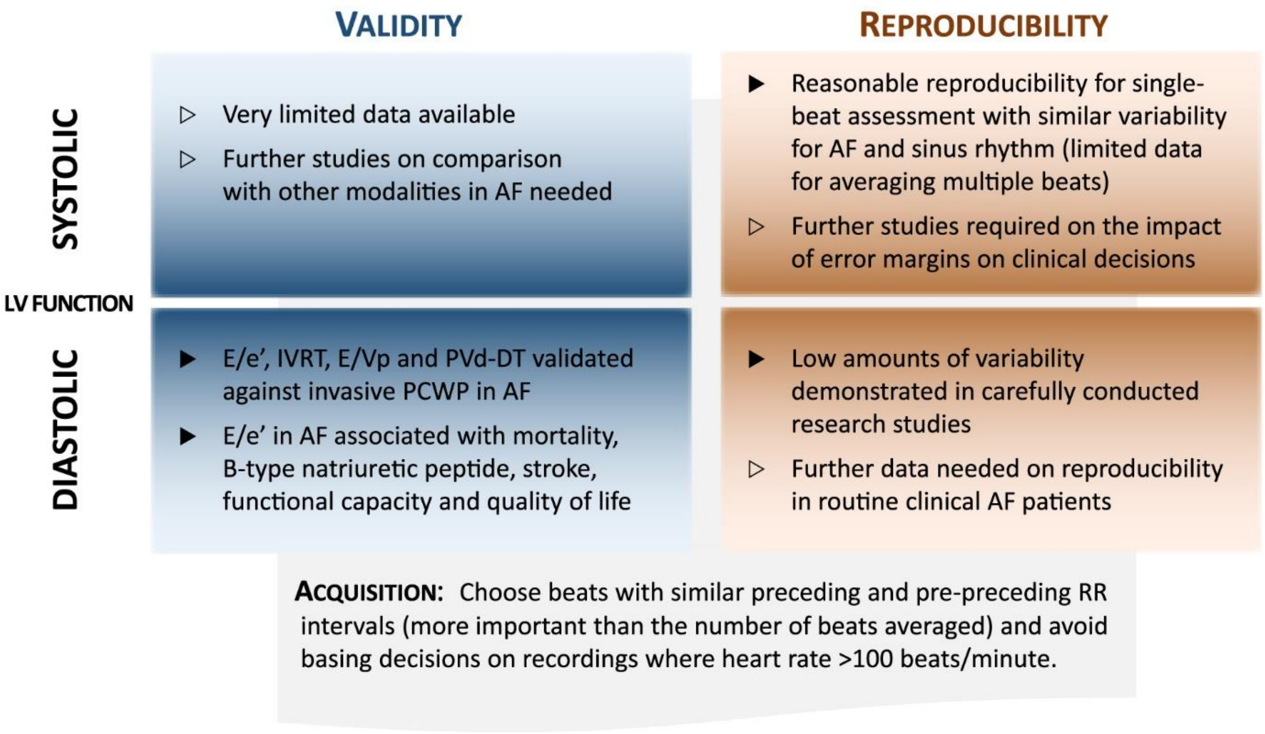
VALIDITY
REPRODUCIBILITY
SYSTOLIC
LV FUNCTION
DIASTOLIC
▷ Very limited data available
▷ Further studies on comparison with other modalities in AF needed
▶ Reasonable reproducibility for single-beat assessment with similar variability for AF and sinus rhythm (limited data for averaging multiple beats)
▷ Further studies required on the impact of error margins on clinical decisions
▶ E/e', IVRT, E/Vp and PVd-DT validated against invasive PCWP in AF
▶ E/e' in AF associated with mortality, B-type natriuretic peptide, stroke, functional capacity and quality of life
▶ Low amounts of variability demonstrated in carefully conducted research studies
▷ Further data needed on reproducibility in routine clinical AF patients
ACQUISITION: Choose beats with similar preceding and pre-preceding RR intervals (more important than the number of beats averaged) and avoid basing decisions on recordings where heart rate >100 beats/minute.

Representative Figure (figure 2) eps | Click here to download Representative Figure Fig2.eps

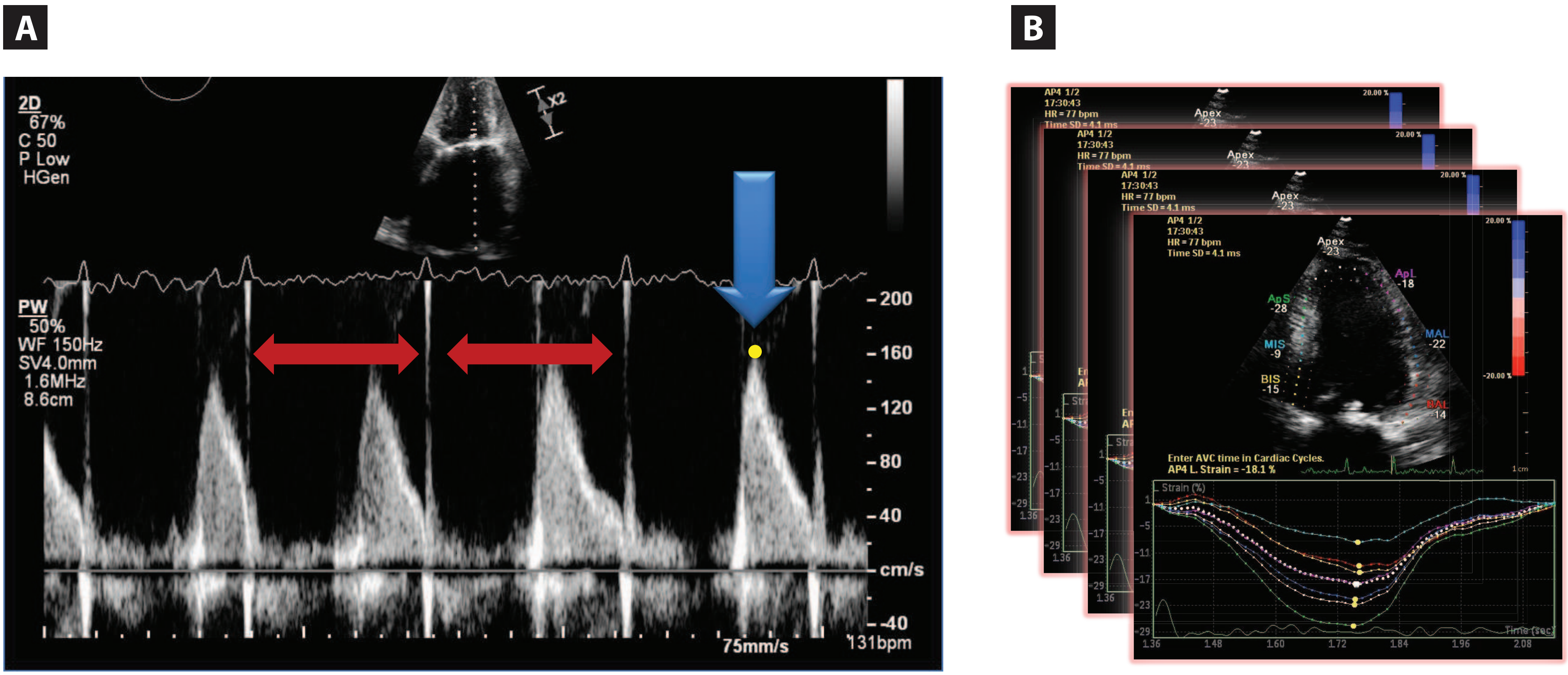

Figure 1 eps

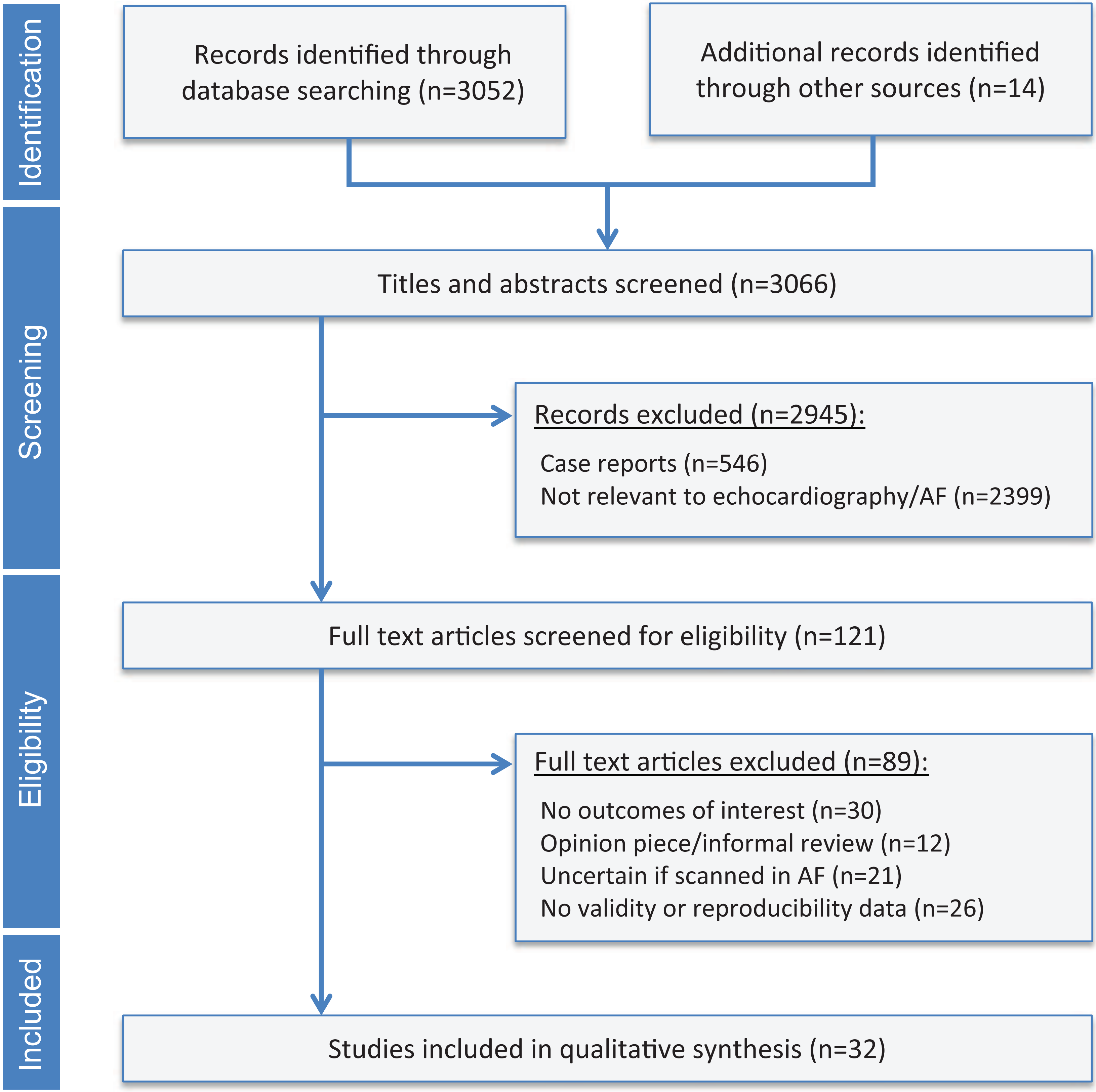
Identification
Screening
Eligibility
Included
Records identified through database searching (n=3052)
Additional records identified through other sources (n=14)
Titles and abstracts screened (n=3066)
Records excluded (n=2945):
Case reports (n=546)
Not relevant to echocardiography/AF (n=2399)
Full text articles screened for eligibility (n=121)
Full text articles excluded (n=89):
No outcomes of interest (n=30)
Opinion piece/informal review (n=12)
Uncertain if scanned in AF (n=21)
No validity or reproducibility data (n=26)
Studies included in qualitative synthesis (n=32)

Figure 3 eps

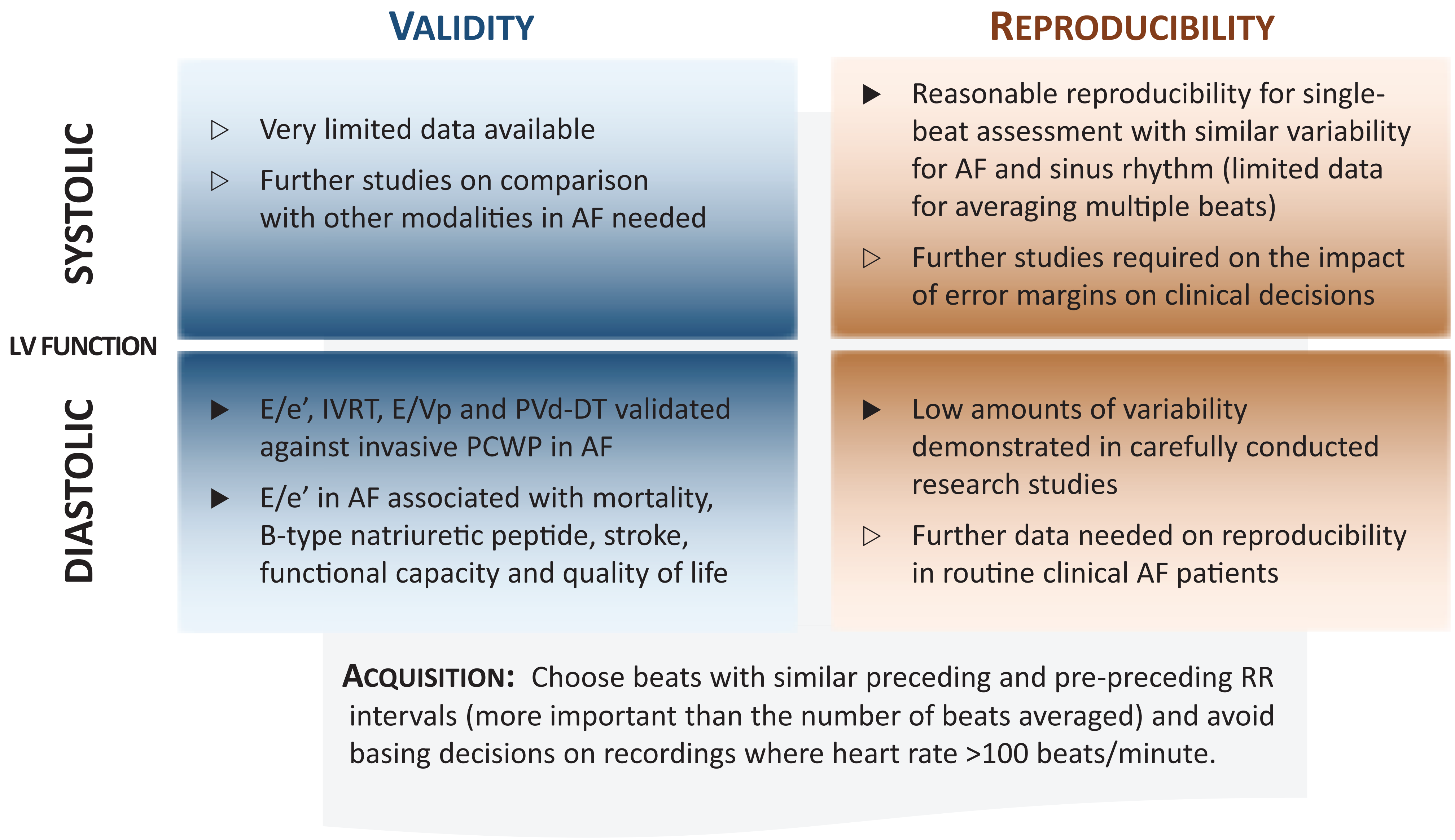
VALIDITY
REPRODUCIBILITY
SYSTOLIC
LV FUNCTION
DIASTOLIC
▷ Very limited data available
▷ Further studies on comparison with other modalities in AF needed
▶ Reasonable reproducibility for single-beat assessment with similar variability for AF and sinus rhythm (limited data for averaging multiple beats)
▷ Further studies required on the impact of error margins on clinical decisions
▶ E/e', IVRT, E/Vp and PVd-DT validated against invasive PCWP in AF
▶ E/e' in AF associated with mortality, B-type natriuretic peptide, stroke, functional capacity and quality of life
▶ Low amounts of variability demonstrated in carefully conducted research studies
▷ Further data needed on reproducibility in routine clinical AF patients
ACQUISITION: Choose beats with similar preceding and pre-preceding RR intervals (more important than the number of beats averaged) and avoid basing decisions on recordings where heart rate >100 beats/minute.
